# Supplementary material for: A comprehensive overview of genomic imprinting in breast and its deregulation in cancer
Source: Nat Commun. 2018 Oct 8;9:4120. doi: 10.1038/s41467-018-06566-7 (PMC6175939; doi:10.1038/s41467-018-06566-7)
Supplement: Supplementary file 1 — Supplementary Information [file 41467_2018_6566_MOESM1_ESM.pdf]

Supplementary Information with

A COMPREHENSIVE OVERVIEW OF GENOMIC IMPRINTING IN BREAST AND ITS DEREGULATION IN CANCER

Goovaerts *et al.*

Supplementary Note 1. SNP tracing & data filtering

After SNP calling and filtering of SNP sites not included in dbSNP, a total of 337,120 SNPs were retained for the 113 normal breast samples. Additional filtering on model fit, FDR-adjusted p-value and median imprinting (see Methods, Section 2) led to a remaining 23,190, 16,214 and 148 SNPS, respectively. The number of SNPs per chromosome and in total are shown in Supplementary Table 1 for these different filtering steps. 140 SNPs (including various *HLA*, *HLA-DR* and *HLA-DQ* genes) were detected as putatively imprinted. Note that sex chromosomes were not taken into account in our analysis. After GTEx validation, 121 SNPs (with annotation) remained.

Supplementary Table 1 Number of SNP positions found after SNP calling that overlapped with SNV positions of dbSNP (Overlap dbSNP), the number of retained loci after prior filtering (fSNPs), additional filtering on (i) FDR-adjusted p-value (adjP SNPs) and (ii) median imprinting (miSNPs) as well as the number of significantly imprinted SNPs (iSNPs). The final set of (annotated) imprinted SNPs (viSNP, validated imprinted SNPs) is shown in the last column.

Chr	Overlap dbSNP	fSNPs	adjP SNPs	miSNPs	iSNPs	viSNP
1	38,200	2,290	1653	5	2	2
2	26,873	1,588	1079	6	6	6
3	23,093	1,505	1064	2	2	1
4	16,806	977	682	4	3	2
5	17,778	1,166	845	1	1	1
6	24,094	1,338	913	20	20	7
7	19,472	1,268	898	6	5	5
8	13,816	871	660	0	0	0
9	17,025	998	642	1	1	0
10	16,627	981	683	1	0	0
11	20,130	1,210	860	13	13	13
12	18,417	1,221	874	1	1	0
13	7,104	426	312	0	0	0
14	13,828	840	551	26	26	26
15	12,239	819	538	29	29	29
16	15,537	967	687	7	6	5
17	20,071	1,286	921	5	5	5
18	6,091	455	315	0	0	0
19	23,714	1,377	888	13	12	12
20	9,983	656	445	7	7	6
21	5,948	374	277	0	0	0
22	10,274	577	427	1	1	1
TOTAL	377,120	23,190	16,214	148	140	121

For putatively imprinted SNPs located in overlapping genes, a rule of thumb was applied to determine the corresponding imprinted gene. As we are assessing RNA-seq data, the most likely imprinted gene is the one in which the SNP is located in an exonic region. However, some loci were not situated in an exon, forcing us to evaluate other regions as well. We hence used the following rule to determine the matching gene for a SNP: exon (coding) > UTR > intron > upstream gene variant/downstream. Applying this rule on the Ensembl annotation of the 121 putatively imprinted SNPs linked the 121 SNPs to 30 genes.

Supplementary Note 2. Mixture distributions of putatively imprinted genes (TCGA)

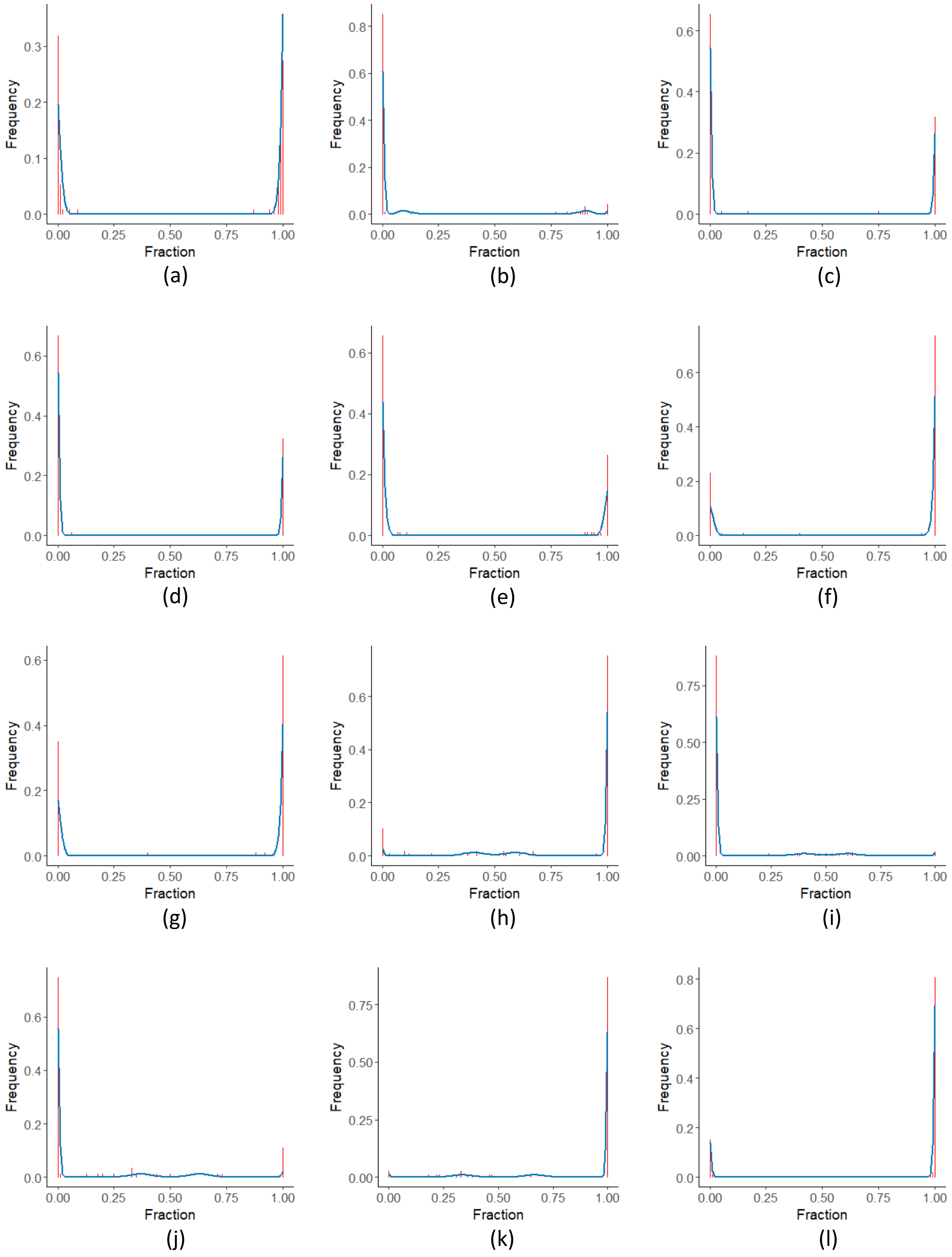

Supplementary Fig. 1 Mixture distributions of significantly imprinted SNPs. (a) rs112232512 (*MTCO1P12*, adj. p-value = 1.42E-75, candidate imprinted) (b) rs61746209 (*LINC01139*, adj. p-value = 6.14E-29) (c) rs7582864 (*ZDBF2*, adj. p-value = 1.02E-21) (d) rs3732084 (*ZDBF2*, adj. p-value = 6.54E-26) (e) rs1975597 (*ZDBF2*, adj. p-value = 7.97E-30) (f) rs1448902 (*ZDBF2*, adj. p-value =2.02E-17) (g) rs4673350 (*ZDBF2*, adj. p-value = 2.56E-23) (h) rs7585510 (*PAX8-AS1*, adj.p-value = 0.0068) (i) rs73158510 (*PTX3*, adj. p-value = 5.44E-07, candidate imprinted) (j) rs12497062 (*CPHL1P*, adj. p-value = 0.00027, filtered – not validated in GTEx) (k) rs11940243 (*ATP8A1*, adj. p-value = 3.21E-08, filtered – not validated in GTEx) (l) rs8605 (*NAP1L5*, adj. p-value = 3.77E-22)

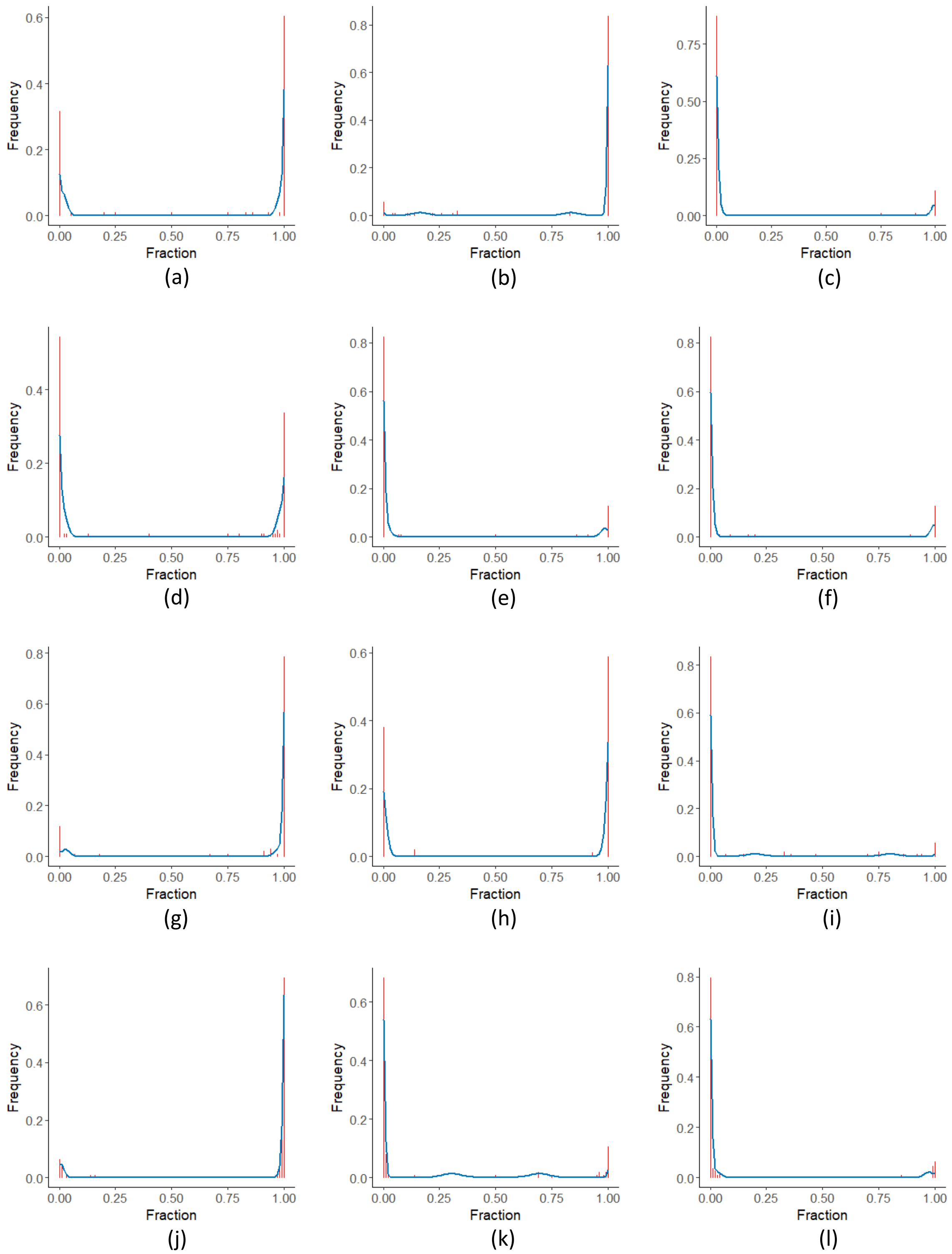

Supplementary Fig. 2 Mixture distributions of significantly imprinted SNPs. (a) rs710834 (*NAP1L5*, adj. p-value = 9.10E-21) (b) rs17800987 (*ZNF300P1*, adj. p-value = 4.87E-11) (c) rs2328535 (*PLAGL1*, adj. p-value = 3.09E-10) (d) rs9373409 (*PLAGL1*, adj. p-value = 1.40E-27) (e) rs73006222 (*PLAGL1*, adj. p-value = 2.41E-15) (f) rs17615967 (*PLAGL1*, adj. p-value = 1.76E-14) (g) rs77203559 (*PLAGL1*, adj. p-value = 3.76E-15) (h) rs9321953 (*PLAGL1*, adj. p-value = 1.48E-21) (i) rs241407 (*LOC100294145*, adj. p-value = 2.71E-07) (j) rs35237090 (*PEG10*, adj. p-value = 1.69E-17) (k) rs13073 (*PEG10*, adj. p-value = 3.35E-13) (l) rs7810469 (*PEG10*, adj. p-value = 6.18E-29)

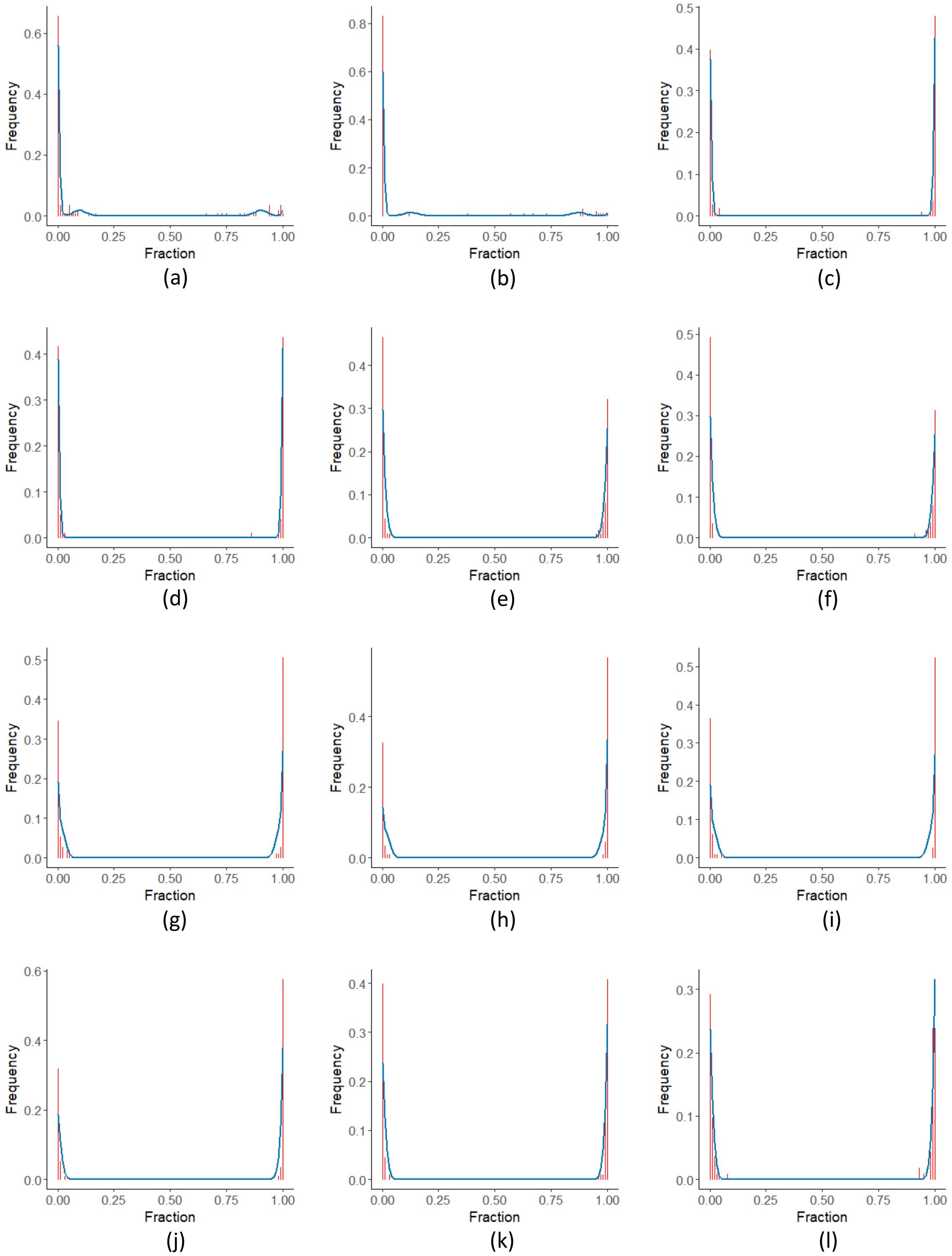

Supplementary Fig. 3 Mixture distributions of significantly imprinted SNPs. (a) rs10863 (*MEST*, adj. p-value = <detection limit) (b) rs706018 (*HOTAIRM1*, adj. p-value = 5.67E-36) (c) rs2075745 (*H19*, adj. p-value = 6.63E-34) (d) rs2075744 (*H19*, adj. p-value = 3.21E-27) (e) rs2839698 (*H19*, adj. p-value = 3.18E-57) (f) rs2067051 (*H19*, adj. p-value = 1.94E-57) (g) rs2839701 (*H19*, adj. p-value = 3.29E-61) (h) rs2839704 (*H19*, adj. p-value = 1.40E-44) (i) rs2839702 (*H19*, adj. p-value = 2.94E-50) (j) rs2839703 (*H19*, adj. p-value = 1.10E-46) (k) rs10840159 (*H19*, adj. p-value = 2.28E-50) (l) rs3741219 (*H19*, adj. p-value = 1.01E-107)

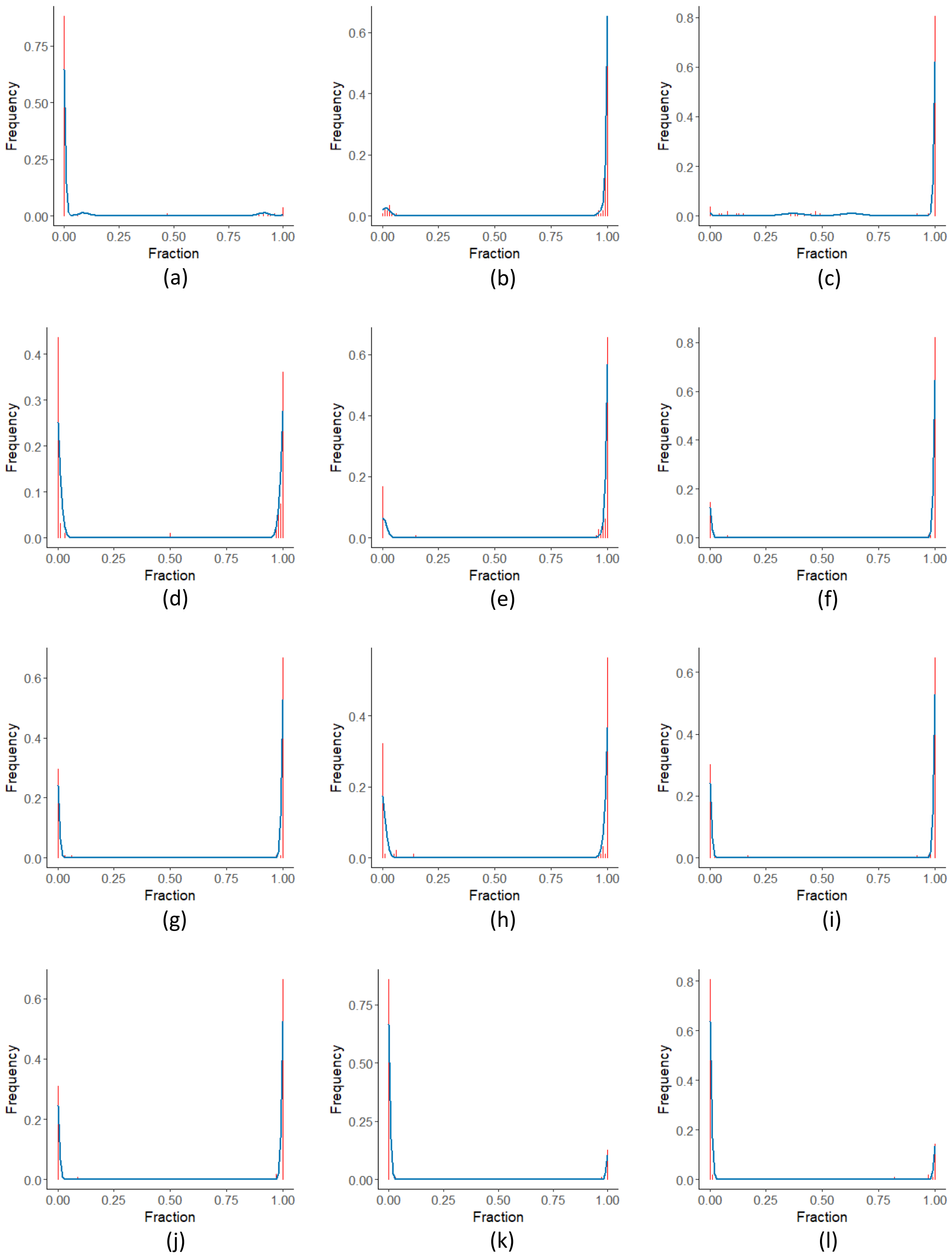

Supplementary Fig. 4 Mixture distributions of significantly imprinted SNPs. (a) rs201284359 (*RP11-109L13.1*, adj. p-value = 4.97E-10, candidate imprinted) (b) rs7873 (*IGF2*, adj. p-value = 1.08E-268) (c) rs1056905 (*GLIPR1/KRR1*, adj. p-value = 1.20E-06, filtered – not validated in GTEx) (d) rs1802710 (*DLK1*, adj. p-value = 2.68E-28) (e) rs78793760 (*MEG3*, adj. p-value = 4.16E-21) (f) rs35458454 (*MEG3*, adj. p-value = 5.16E-21) (g) rs35431412 (*MEG3*, adj. p-value = 1.62E-29) (h) rs10147988 (*MEG3*, adj. p-value = 1.07E-26) (i) rs3087918 (*MEG3*, adj. p-value = 5.47E-27) (j) rs3087917 (*MEG3*, adj. p-value = 1.64E-30) (k) rs3742391 (*MEG3*, adj. p-value = 2.13E-19) (l) rs12897172 (*MEG3*, adj. p-value = 2.31E-17)

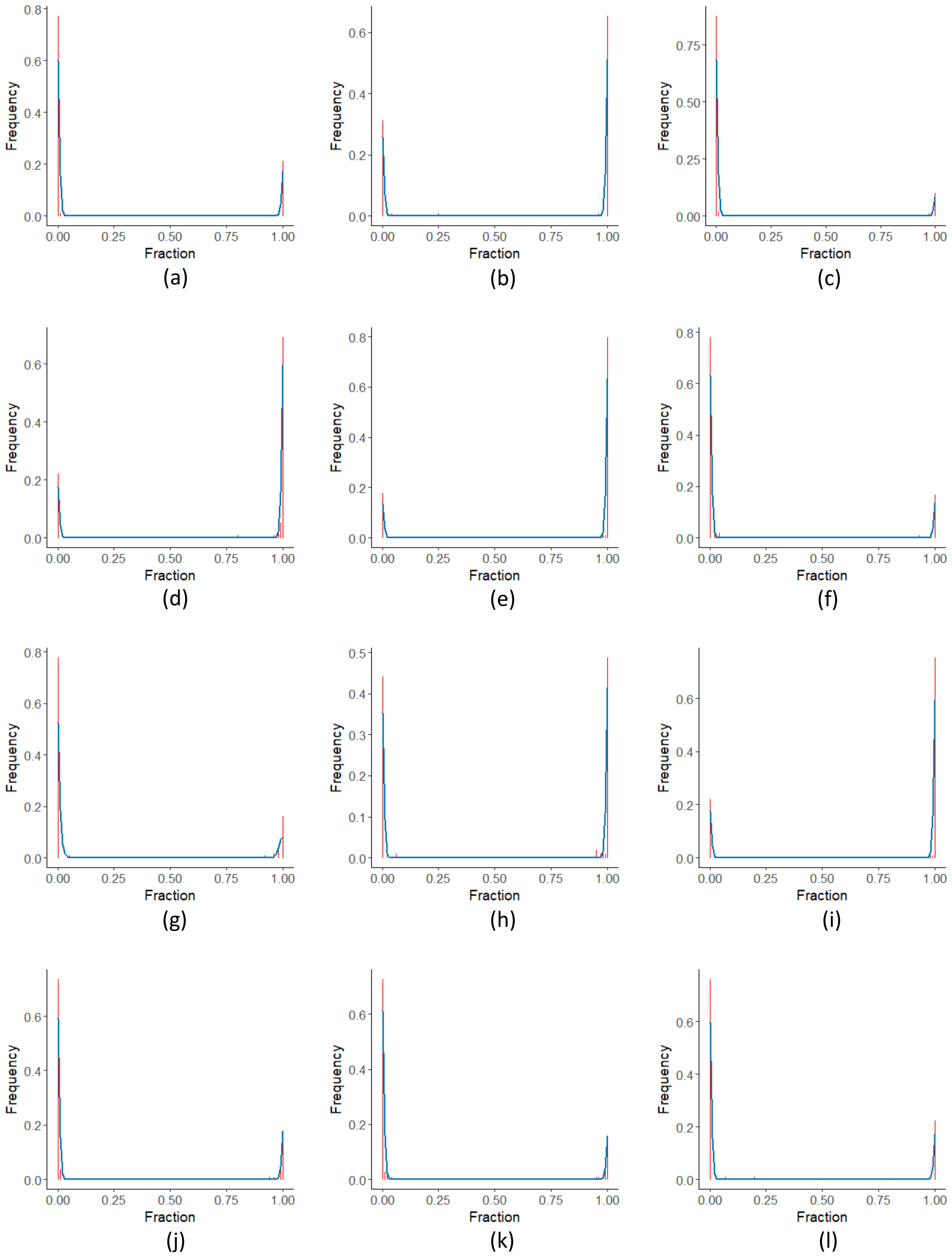

Supplementary Fig. 5 Mixture distributions of significantly imprinted SNPs. (a) rs1884540 (*MEG3*, adj. p-value = 1.78E-26) (b) rs2400941 (*MEG3*, adj. p-value = 1.64E-29) (c) rs77658190 (*MEG3*, adj. p-value = 2.45E-17) (d) rs10132552 (*MEG3*, adj. p-value = 4.26E-17) (e) rs3194464 (*MEG3*, adj. p-value = 1.43E-23) (f) rs11160606 (*MEG3*, adj. p-value = 1.53E-23) (g) rs1950628 (*MEG3*, adj. p-value = 1.01E-26) (h) rs1053900 (*MEG3*, adj. p-value = 4.91E-34) (i) rs1054000 (*MEG3*, adj. p-value = 7.58E-27) (j) rs8013873 (*MEG3*, adj. p-value = 6.63E-27) (k) rs11859 (*MEG3*, adj. p-value = 2.17E-25) (l) rs74080162 (*MEG3*, adj. p-value = 1.15E-24)

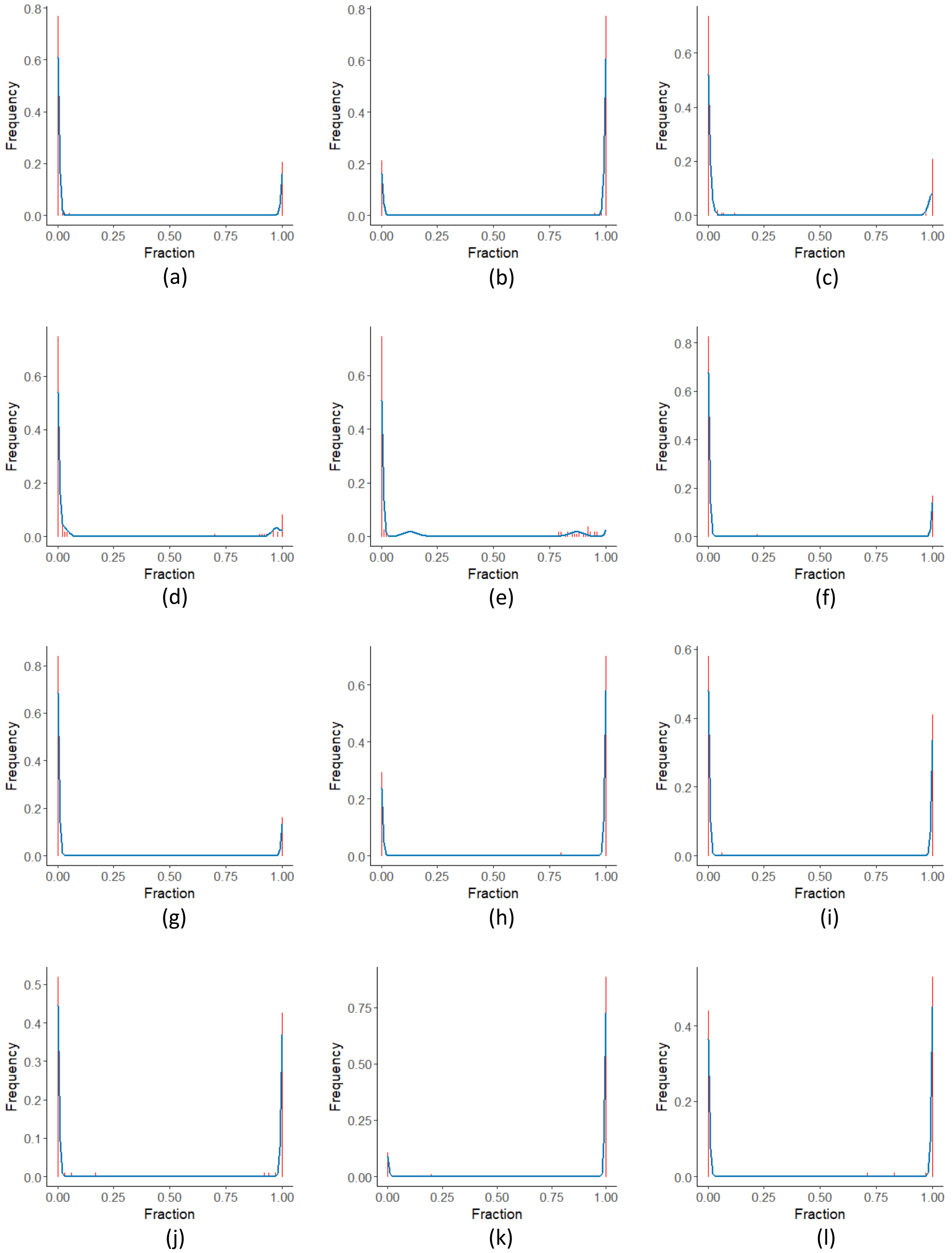

Supplementary Fig. 6 Mixture distributions of significantly imprinted SNPs. (a) rs4378559 (*MEG3*, adj. p-value = 8.76E-25) (b) rs12890215 (*MEG3*, adj. p-value = 8.74E-25) (c) rs55996894 (*MEG3*, adj. p-value = 5.63E-21) (d) rs3742390 (*MEG3*, adj. p-value = 6.24E-31) (e) rs4906022 (*MEG3*, adj. p-value = 8.42E-204) (f) rs2554426 (*SNRPN*, adj. p-value = 9.39E-16) (g) rs2732028 (*SNHG14*, adj. p-value = 3.33E-15) (h) rs74335291 (*SNHG14*, adj. p-value = 5.12E-24) (i) rs2732029 (*SNHG14*, adj. p-value = 6.71E-28) (j) rs765438 (*SNHG14*, adj. p-value = 1.96E-28) (k) rs2732030 (*SNHG14*, adj. p-value = 9.43E-12) (l) rs10451029 (*SNHG14*, adj. p-value = 6.14E-22)

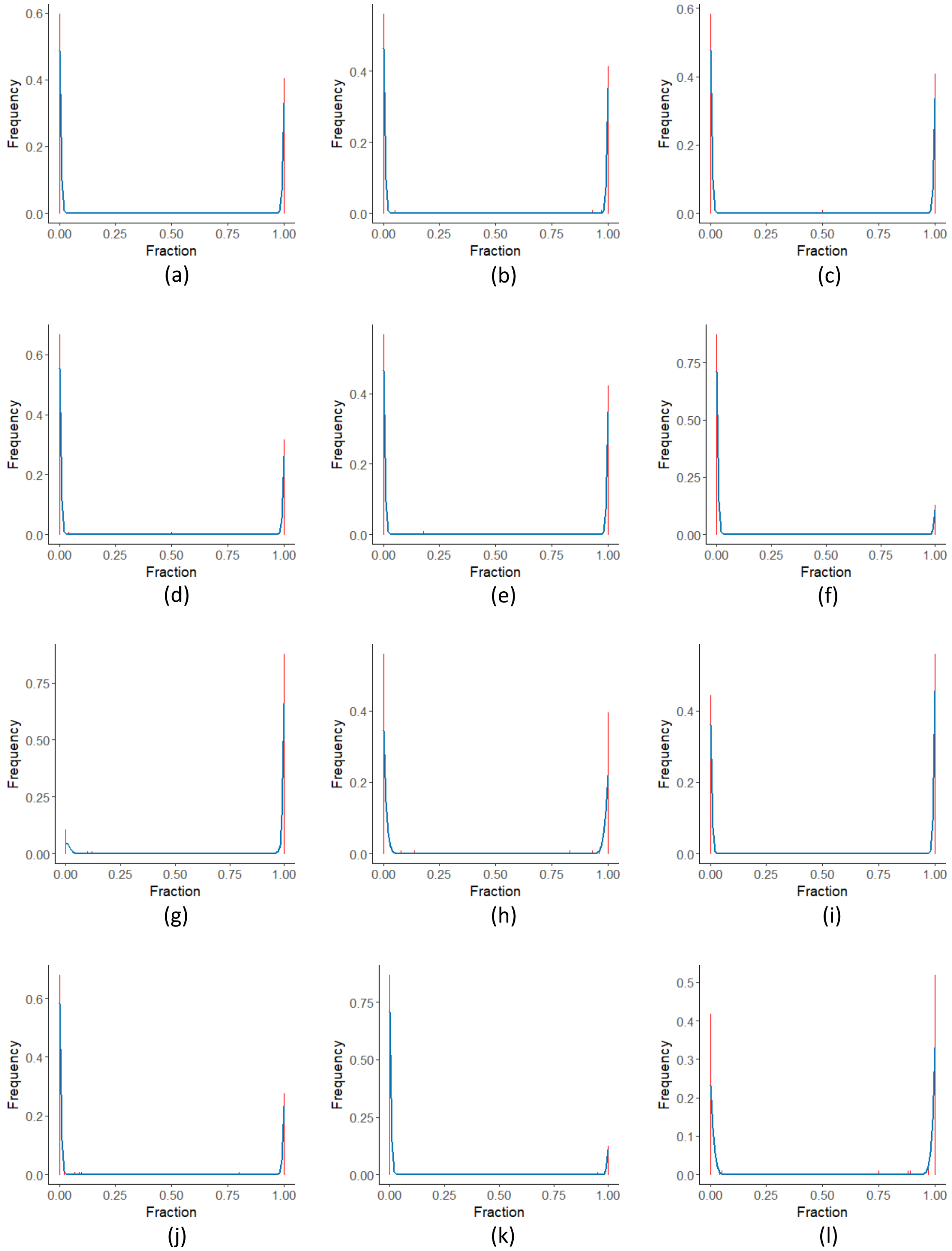

Supplementary Fig. 7 Mixture distributions of significantly imprinted SNPs. (a) rs2052723 (*SNHG14*, adj. p-value = 1.14E-25) (b) rs2554419 (*SNHG14*, adj. p-value = 7.96E-31) (c) rs719704 (*SNHG14*, adj. p-value = 3.32E-22) (d) rs34316840 (*SNHG14*, adj. p-value = 1.13E-25) (e) rs2732031 (*SNHG14*, adj. p-value = 1.23E-26) (f) rs2732041 (*PWAR6*, adj. p-value = 1.60E-12) (g) rs2732043 (*PWAR6*, adj. p-value = 1.78E-13) (h) rs2732044 (*PWAR6*, adj. p-value = 1.42E-26) (i) rs1030389 (*PWAR6*, adj. p-value = 1.68E-28) (j) rs62001981 (*PWAR6*, adj. p-value = 1.41E-26) (k) rs62001982 (*PWAR6*, adj. p-value = 1.09E-19) (l) rs1045935 (*PWAR6*, adj. p-value = 6.76E-29)

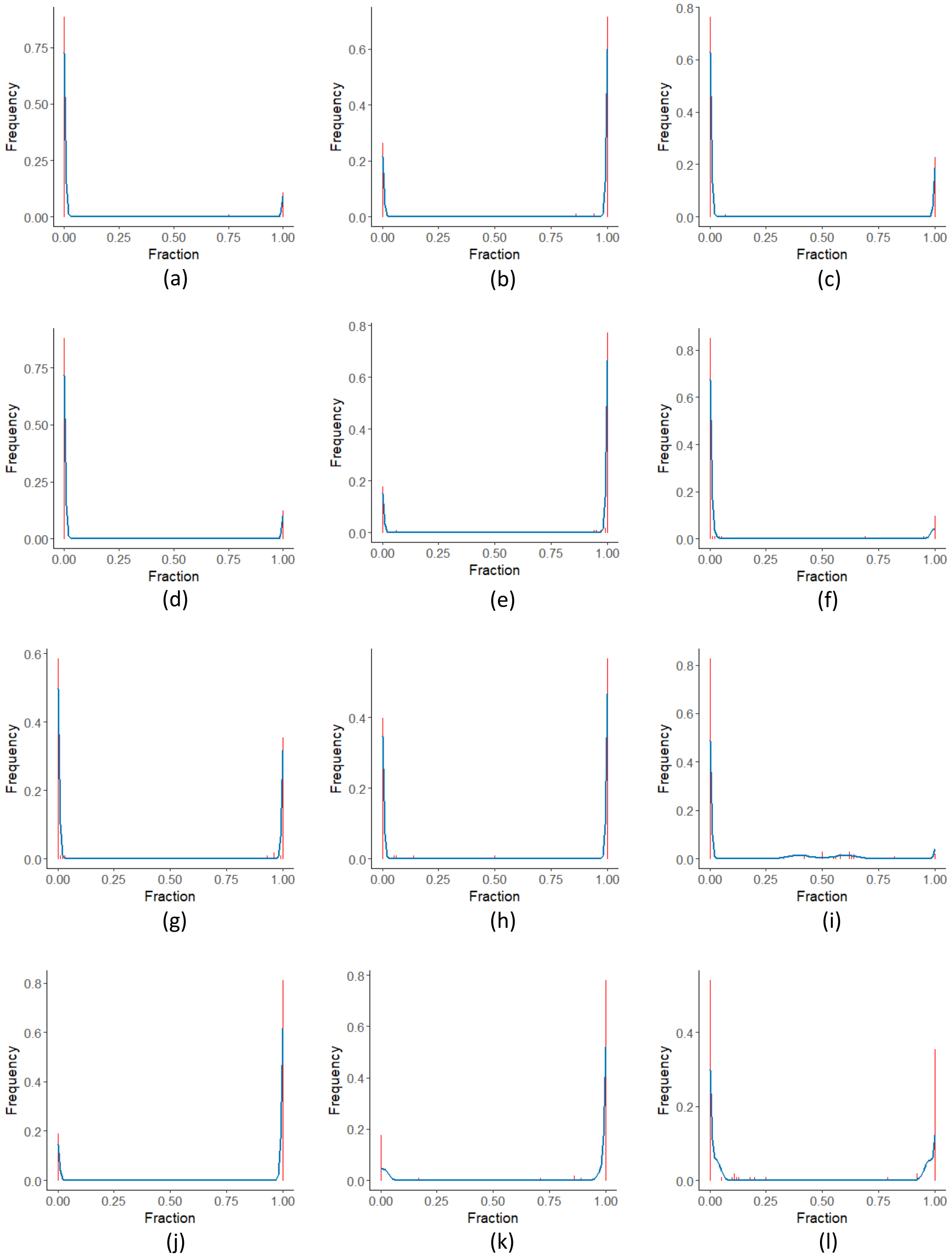

Supplementary Fig. 8 Mixture distributions of significantly imprinted SNPs. (a) rs11637436 (*SNHG14*, adj. p-value = 1.44E-11) (b) rs4344720 (*SNHG14*, adj. p-value = 7.48E-20) (c) rs3863396 (*SNHG14*, adj. p-value = 2.68E-19) (d) rs62002013 (*SNHG14*, adj. p-value = 3.83E-12) (e) rs2356294 (*SNHG14*, adj. p-value = 4.96E-24) (f) rs1043164 (*SNHG14*, adj. p-value = 6.41E-12) (g) rs691 (*SNHG14*, adj. p-value = 6.11E-33) (h) rs13526 (*SNHG14*, adj. p-value = 3.74E-29) (i) rs4578621 (*PLIN1*, adj. p-value = 3.29E-06, candidate imprinted) (j) rs37822 (*ZNF597*, adj. p-value = 1.47E-18) (k) rs37823 (*ZNF597*, adj. p-value = 3.08E-18) (l) rs11639510 (*ZNF597*, adj. p-value = 3.42E-25)

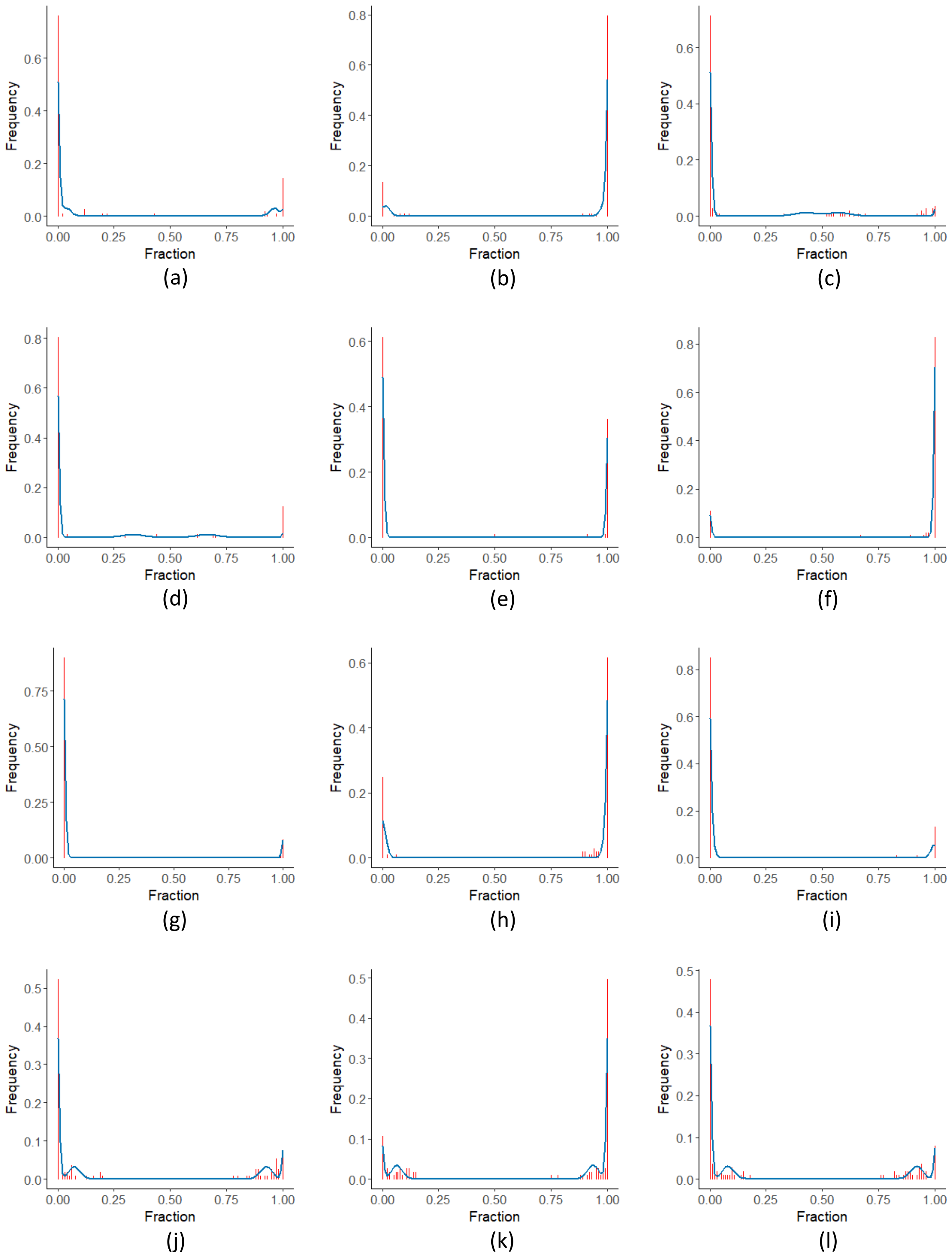

Supplementary Fig. 9 Mixture distributions of significantly imprinted SNPs. (a) rs37824 (*ZNF597*, adj. p-value = 3.21E-18) (b) rs12737 (*ZNF597*, adj. p-value = 1.01E-21) (c) rs77309587 (*TPSB2*, adj. p-value = 1.52E-08, filtered – not validated in GTEx) (d) rs141915702 (*USP32P2*, adj. p-value = 9.30E-03, candidate imprinted) (e) rs3931649 (*MTRNR2L1*, adj. p-value = 3.16E-28, candidate imprinted) (f) rs113014658 (*MTRNR2L1*, adj. p-value = 6.82E-09, candidate imprinted) (g) rs113626706 (*MTRNR2L1*, adj. p-value = 5.74E-12, candidate imprinted) (h) rs3931650 (*MTRNR2L1*, adj. p-value = 7.67E-29, candidate imprinted) (i) rs113983639 (*ZNF331*, adj. p-value = 1.99E-13) (j) rs8110350 (*ZNF331*, adj. p-value = 4.12E-106) (k) rs8110538 (*ZNF331*, adj. p-value = 1.74E-109) (l) rs8109631 (*ZNF331*, adj. p-value = 8.52E-175)

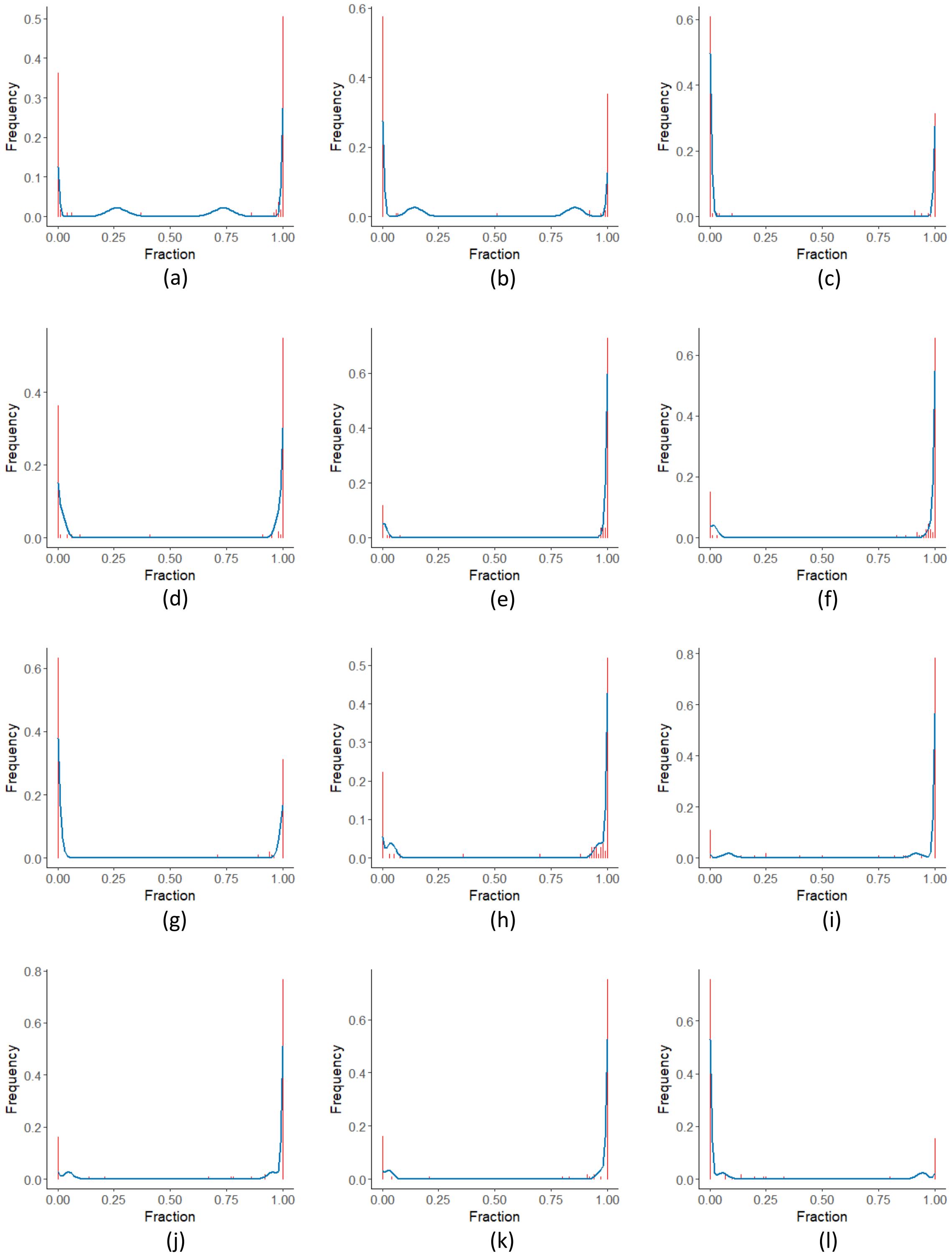

Supplementary Fig. 10 Mixture distributions of significantly imprinted SNPs. (a) rs4801386 (*PEG3*, adj. p-value = 1.10E-04) (b) rs1558355 (*PEG3*, adj. p-value = 2.70E-04) (c) rs723082 (*PEG3*, adj. p-value = 1.62E-31) (d) rs3143 (*PEG3*, adj. p-value = 7.86E-06) (e) rs1055359 (*PEG3*, adj. p-value = 9.07E-20) (f) rs11666110 (*PEG3*, adj. p-value = 8.06E-28) (g) rs1860565 (*PEG3*, adj. p-value = 1.02E-21) (h) rs33931963 (*PEG3*, adj. p-value = 6.73E-28) (i) rs6058058 (*HM13*, adj. p-value = 1.33E-10) (j) rs6059869 (*HM13*, adj. p-value = 4.04E-14) (k) rs6059873 (*HM13*, adj. p-value = 1.29E-19) (l) rs6059874 (*HM13*, adj. p-value = 4.88E-14)

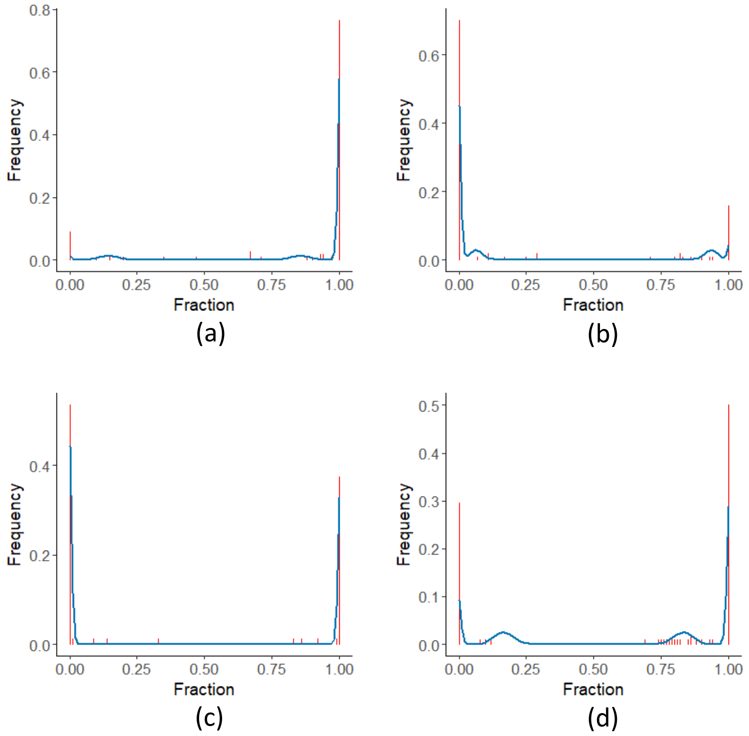

Supplementary Fig. 11 Mixture distributions of significantly imprinted SNPs. (a) rs1115713 (*HM13*, adj. p-value = 7.94E-09) (b) rs2269621 (adj. p-value = 1.84E-18, filtered (no dbSNP annotation, though likely *L3MBTL1*)) (c) rs1800900 (*GNAS/GNAS-AS1*, adj. p-value = 5.02E-16) (d) rs550197 (*BCR*, adj. p-value = 7.93E-50)

Supplementary Note 3. Validation of putatively imprinted regions

a. Comparison with external references

To independently validate our methodology, we compared these loci with the results found by Baran *et al.*[1]. By using RNA-seq data combined with genotype data from 27 primary breast tissue samples (GTEx project), they were able to establish (partial) imprinting in breast tissue in 15 genes, including both novel and previously identified genes (Supplementary Data5, see[1]). They also detected several HLA genes, but as already stated in the Results section, these are not taken into account. In summary, 13 genes were also found to be imprinted in breast tissue by our methodology, whereas 1 (= *PPIEL*) was not detected due to too low coverage in our samples (i.e. was filtered out prior to statistical analysis). For a second gene, *SNURF*, intronic SNPs were detected to be imprinted in TCGA as well. Yet, based on manual curation, it was deemed more likely that *SNRPN* was the imprinted gene rather than *SNURF*. Of the 23 genes not identified by Baran *et al.* as imprinted, 5 have been identified as imprinted in blood by Joshi et *al.*[2]. More genes concurred between the latter method and ours, however, many were curated due to annotation difficulties. Also, several genes were located near known imprinted regions. For example, *PWAR6* is a gene lying in the Prader-Willi/Angelman region (PWAR), a region on chromosome 15 for which deregulation of the imprinting pattern is known to result in imprinting disorders, i.e. Prader-Willi and Angelman syndromes. Also *SNHG14* is found in this region.

An additional comparison was made with Geneimprint - a species-specific database containing already known imprinted or predicted imprinted genes with some (preliminary) evidence over all tissues – and the Imprinted Genes Catalogue – a database containing genes showing parent-of-origin effects[3,4]. Our methodology successfully detected 17 of these genes (with 2 genes found in only one of the

databases) to be monoallelically expressed in the 113 normal breast tissue samples (coloured in green or orange in Supplementary Table 2). Though 19 genes were not yet included in Geneimprint, it should be noted that for several genes, alternative independent evidence for imprinting is available.

Supplementary Table 2 Overlap between our detected imprinted SNPs and the Geneimprint[3] and Imprinted Genes Catalogue[4]. The genes found in both databases and detected by our method are coloured in green. In red imprinted genes are shown which are not found in either of the databases. Genes found in one of the databases are coloured orange.

*MTCO1P12***	*LINC01139*	*ZDBF2*	*PAX8-AS1*	*PTX3***	*CPHL1P**
*ATP8A1**	*NAP1L5*	*ZNF300P1*	*PLAGL1*	*LOC100294145*	*PEG10*
MEST	*HOTAIRM1*	*H19*	*RP11-109L13.1***	*IGF2*	*GLIPR1**
*KRR1**	*DLK1*	*MEG3*	*SNRPN*	*SNHG14*	*PWAR6*
*PLIN1***	*ZNF597*	*TPSB2**	*USP32P2***	*MTRNR2L1***	*ZNF331*
PEG3	*HM13*	*L3MBTL1*	*GNAS-AS1*	*GNAS*	*BCR*

Genes eliminated after GTEx validation are indicated with * and candidate imprinted genes with **.

b. Comparison with previously found monoallelically methylated loci

In 2014, our lab created a data-analytical framework based on the Hardy-Weinberg theorem that - using enrichment-based sequencing data (e.g. MethylCap-seq) - screens for regions featured by monoallelic DNA methylation in a genome-wide manner[5]. Applied on 334 MethylCap-seq samples of diverse origin, 80 genomic regions were identified that featured significant monoallelic methylation, of which 49 were located in genic regions. As an additional evaluation, these results were combined with the here reported monoallelically expressed genes (Supplementary Table 3).

Supplementary Table 3 Overlap between monoallelically methylated regions found by Steyaert *et al.* and the imprinted genes identified by the current methodology[5]. Shown are the genes found by the former method. Genes also identified as monoallelically expressed by our framework are coloured in green, whereas genes not detected as imprinted by our method are coloured in red. Genes which were not (sufficiently) covered in our dataset - and thus were not analysed by our framework - are shown in grey.

DUSP5P	*RN5S6*	*ZDBF2*	*ANTXR1*	*CDC27P1*
MFI2	*SORCS2*	*HERC3*	*NAP1L5*	*ADAMTS2*
FAM50B	*RP11-420L9.4.1*	*WDR27*	*AC007091.1.1*	*PTPRN2*
SEC31	*NDUFB8*	*KCNQ1*	*KCNQ10T1*	*MIR675*
H19	*C13ORF33*	*LINC00351*	*RP11-1152H15.1.1*	*SNURF*
SNRPN	*NAA60*	*ZNF597*	*RMI2*	*AC136932.1*
ACCN1	*PEG3*	*ZIM2*	*NOTCH3*	*AC092279.1*
ZNF331	*GNAS-AS1*	*GNAS*	*TMPRSS3*	*WRB*
MX2	*FAM19A5*	*NHP2L1*		

The presence of genes overlapping between both datasets suggests that monoallelic methylation of these loci is associated with - and is possibly involved in the mechanism of - the imprinted expression pattern. Here, 8 overlapping genes were found featuring both monoallelic methylation as well as monoallelic expression.

c. Methodological validation using whole exome sequencing data

The reliability of using RNA-seq data without corresponding DNA sequencing inferred genotypes for detection of imprinting was tested by comparing RNA-seq based genotypes with WES based genotypes (both SeqEM inferred) on the control data. Basically, it was evaluated whether for imprinted SNPs, heterozygous samples (as identified by WES) were indeed featured by monoallelic expression (RNA-seq). As already mentioned, inclusion of whole exome sequencing data for validation

of imprinted SNPs led to massive loss of data. The WES data often showed too low coverage for accurate genotyping. Increasing the minimal reads threshold resulted in loss of many of the called genotypes (Supplementary Fig. 12). Many of our detected SNPs are located in intronic regions or UTR's, which could explain the low coverage or absence of the SNPs in WES data. As the minimal coverage imposed for genotyping increases, however, the concordance between the genotypes called by RNA-seq and WES reaches a plateau phase. Although only a limited set of genotypes remained after enforcing a minimal reads threshold, we were able to confirm imprinting in the control data for virtually all heterozygous SNPs (Supplementary Fig. 13 and Supplementary Table 4). We can hence conclude that for data with adequate quality, RNA-seq suffices for the detection of imprinting.

Subsequently, we analysed how accurate the allelic ratio is for determining heterozygous samples featured by biallelic expression in RNA-seq data of the tumour samples. By increasing the minimal allelic ratio for calling a sample biallelically expressing (and thus heterozygous), the concordance between RNA-seq and WES increased as well (Supplementary Fig. 14). Low allelic ratios probably represent sequencing errors in the RNA-seq data, yet some SNPs remained discordant even after raising the reads threshold for both WES and RNA-seq. After looking into more detail in those SNPs, we found that – next to some remaining sequencing errors/low coverage artefacts – these discordances were probably due to too low minor allele frequencies deluding the SeqEM statistical methodology, though in some rare occasions, software errors may provide a likely alternative explanation.

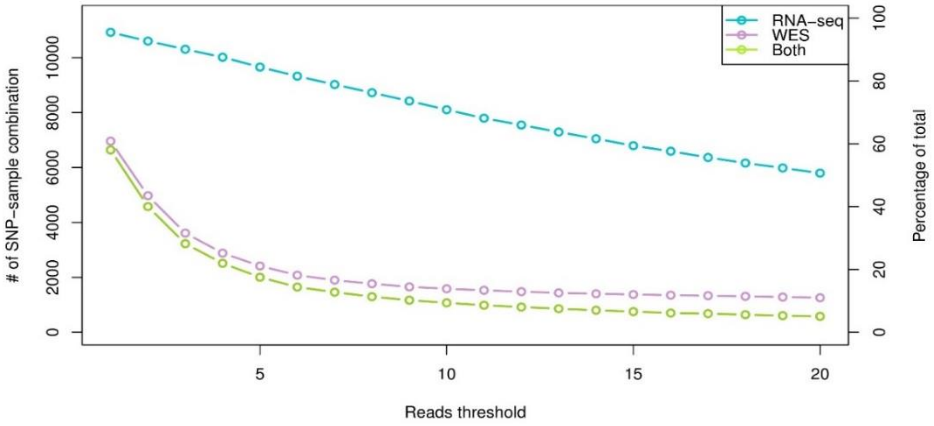

Supplementary Fig. 12 Correspondence between RNA-seq and whole exome sequencing data for the putatively imprinted SNPs of the control samples. As the minimal number of reads increases, the number of SNPs and samples available in whole exome sequencing data decreases and often too few data are available for accurate methodological validation.

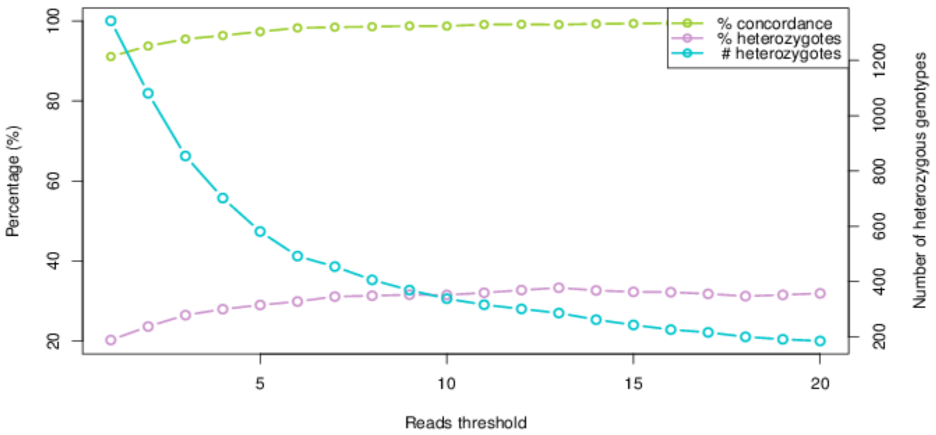

Supplementary Fig. 13 Concordance between the RNA-seq genotypes and whole exome sequencing (WES) genotypes in control samples. As the reads threshold increases (for both RNA-seq and WES), the concordance also increases, but the absolute number of heterozygous loci decreases.

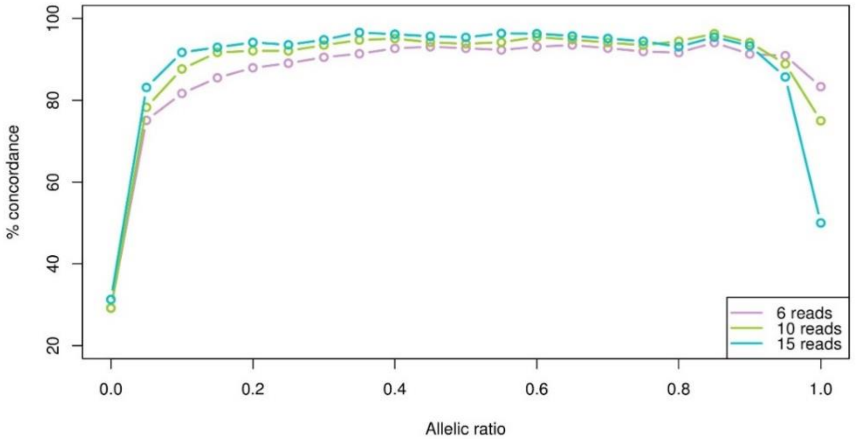

Supplementary Fig. 14 Concordance of determining heterozygous samples in tumour RNA-seq data by allelic ratio with genotypes determined by whole exome sequencing data for varying allelic ratios and minimal reads thresholds. Only few loci had an allelic ratio approximately equal to 1, yet with one error, explaining the sudden drop at the right part of the figure.

Supplementary Table 4 Validation of imprinting by comparison of RNA "genotype" with whole exome sequencing (WES) data. Concordance is shown between the heterozygous DNA samples that were indeed apparently homozygous on the RNA level. Varying minimal coverages are shown.

Min coverage	Heterozygote DNA	Homozygote RNA	Concordance (%)
5	467	447	95.7
10	275	258	93.8
15	206	191	92.7
20	163	151	92.6

d. Candidate imprinted loci and transcript-specific imprinting

GTEx healthy breast data was used to validate putatively imprinted genes. Validation led to elimination of four genes from our set of imprinted genes that could not be verified in GTEx data. Furthermore, four other genes were denoted as candidate imprinted. We were able to validate these genes in GTEx, but they showed a mixed imprinting pattern in TCGA that could not be explained by transcript-specific imprinting as no consistent results between exons were found (Supplementary Fig. 15). On the other hand, the more complex imprinting patterns of *ZNF300P1*, *LOC100294145*, *ZNF331* and *GNAS/GNAS-AS1* in TCGA showed consistent differences between exons and transcripts (Supplementary Fig. 16), while for *HOTAIRM1* evidence was less clear (Supplementary Fig. 17).

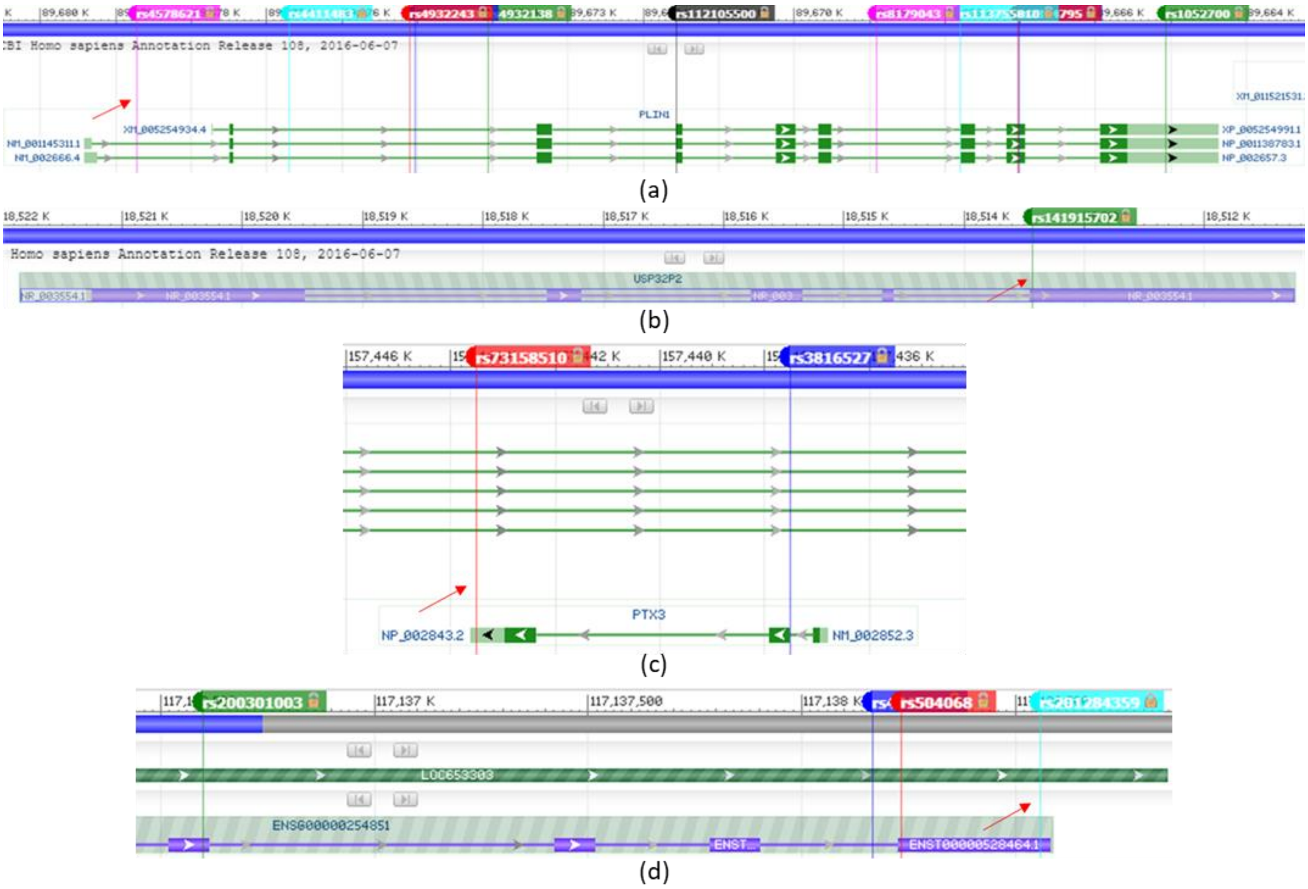

Supplementary Fig. 15 Gene structure from candidate imprinted genes. Transcript maps of four genes denoted as candidate imprinted: (a) *PLIN1*, (b) *USP32P2*, (c) *PTX3* and (d) *RP11-109L13.1*. The red arrow indicates the SNP that was detected as imprinted in TCGA data. The other SNPs were either not detected due to technical issues or were clearly not imprinted.

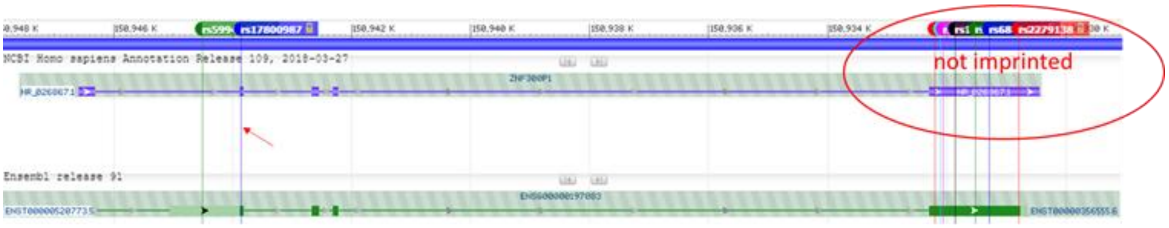

(a)

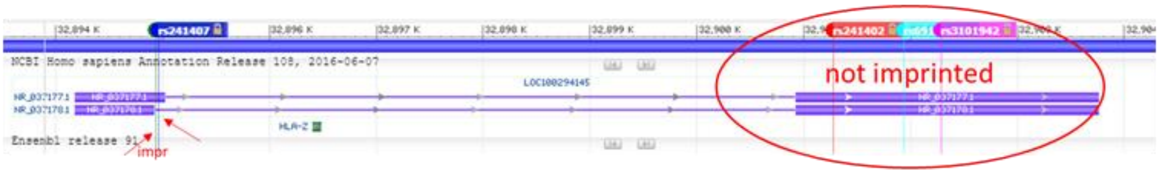

(b)

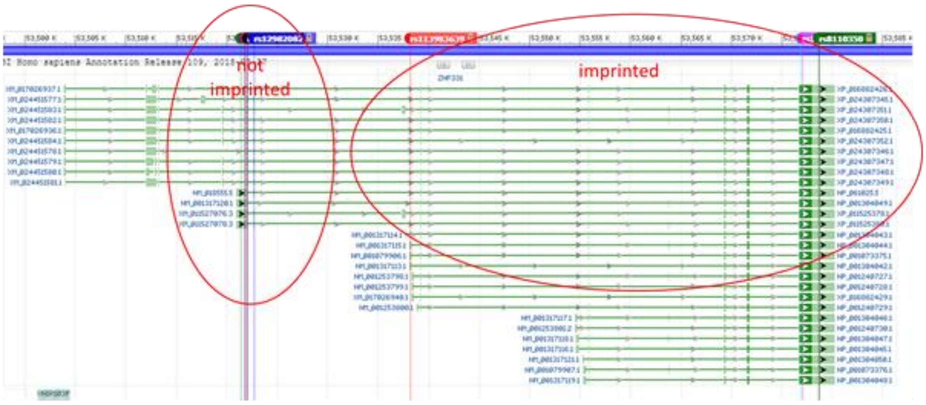

(c)

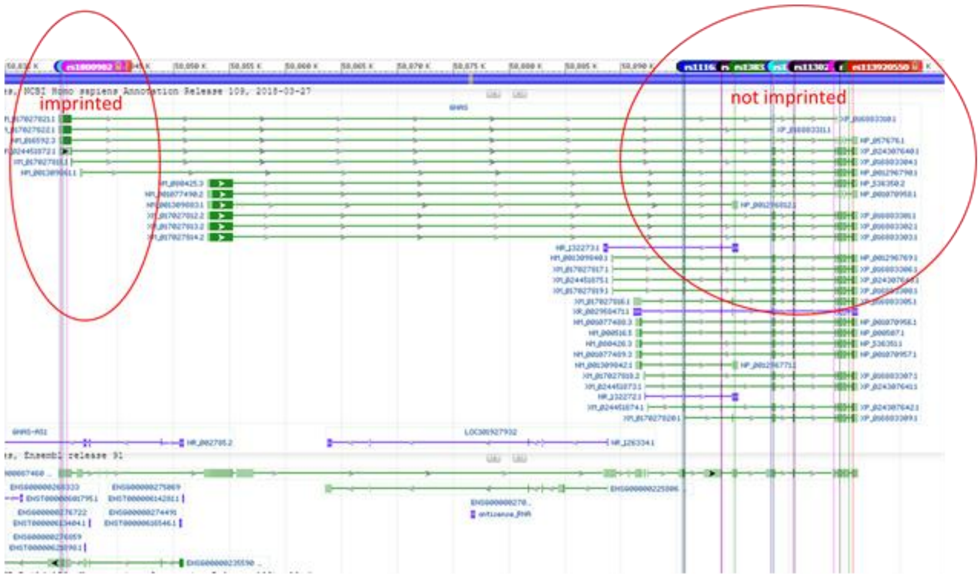

(d)

Supplementary Fig. 16 Gene structure from putative imprinted genes with transcript-specific imprinting patterns. Transcript maps of four genes: (a) *ZNF300P1*, (b) *LOC100294145*, (c) *ZNF331* and (d) *GNAS/GNAS-AS1*. The red arrow/circle indicates the SNPs that were clearly imprinted in TCGA data. The other SNPs were either not detected due to technical issues or were clearly not imprinted. Differences between imprinting patterns could here be attributed to transcript-specific effects.

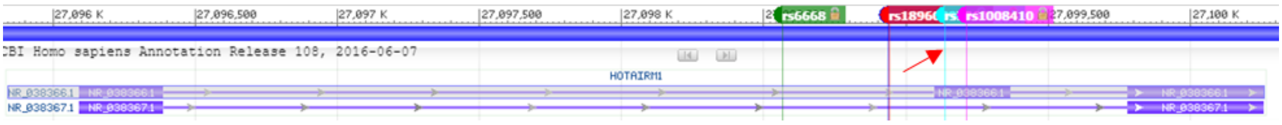

Supplementary Fig. 17 Gene structure of *HOTAIRM1*. The red arrow indicates the SNP that was clearly imprinted in TCGA data. The other SNPs were either not detected due to technical issues or were clearly not imprinted. Differences between imprinting patterns could here be attributed to transcript-specific effects.

To further evaluate whether inconsistencies for SNPs within a single gene are actually due to technical/power associated causes, or due to re-expression of overlapping non-imprinted isoforms, we analysed all (significant and non-significant) SNPs in some additional genes with known overlapping non-imprinted transcripts not discussed higher (*GRB10*, *MEST*, *INPP5F/INPP5Fv2*, *NAPIL5/HERC3*, *PPIEL*, *ZNF597/NAA60/MIR6126*, *WRB*, *SNU13* and *C22orf46*). Most genes for which we did not observe any imprinting (*GRB10*, *INPP5F*, *NAA60*, *WRB*, *SNU13* and *C22orf46*, Supplementary Fig. 18(c) and (e) and Supplementary Fig. 19, respectively) were clearly not imprinted independent of overlapping transcripts. Only *PPIEL* was missed due to technical issues, mainly low coverage for putatively imprinted SNPs. Note that this was also the locus found by Baran *et al.* that we missed.[1] Supplementary Fig. 18(a) illustrates that overlapping transcripts may disguise imprinting, yet the evidence/coverage was too poor to call this an imprinting candidate in our data. For *NAP1L5* and *MEST* imprinting was clear in the actual genes (non-significant SNPs only due to technical issues, Supplementary Fig. 18(b) and (d), respectively), whereas the overlapping genes were certainly not imprinted.

In summary, visual evaluation of loci featured by complex imprinting patterns verifies that we extracted the large majority of (complex) imprinting events clearly present in the data, i.e. the current bottleneck for further improvement is the short-read RNA-seq data rather than the proposed methodology.

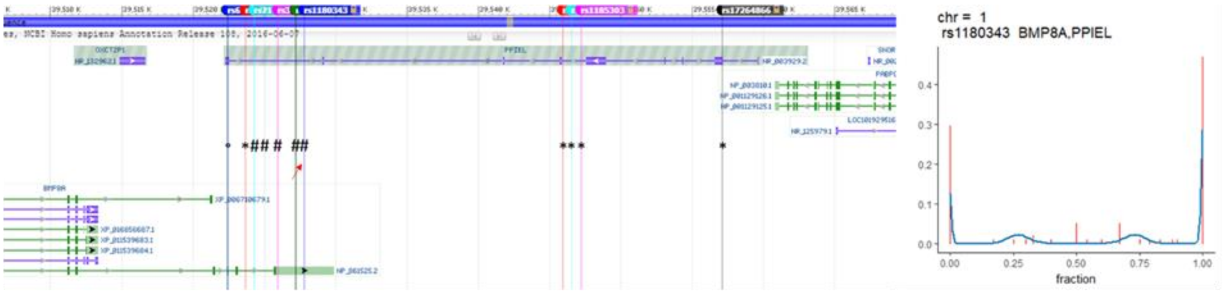

(a)

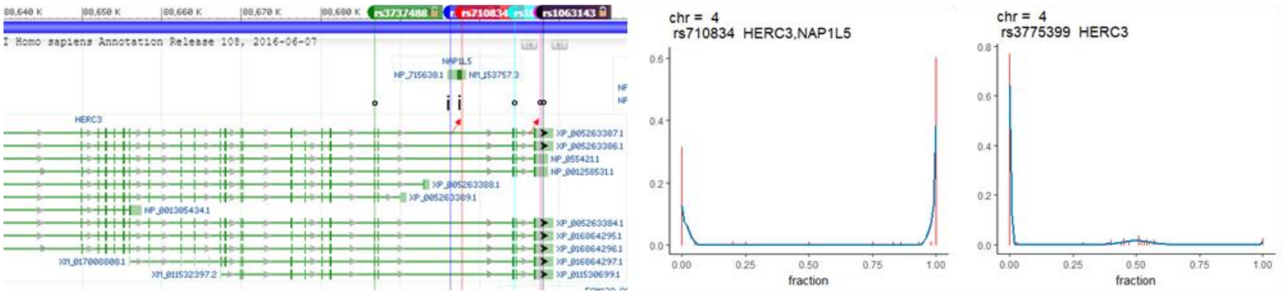

(b)

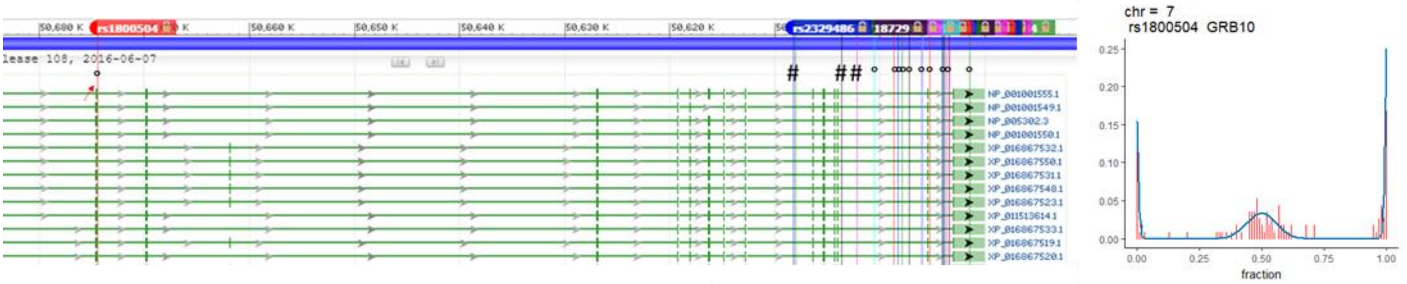

(c)

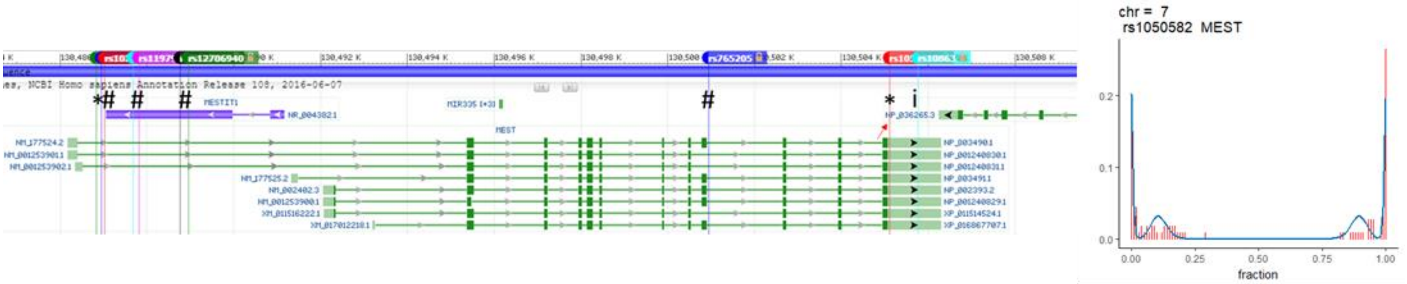

(d)

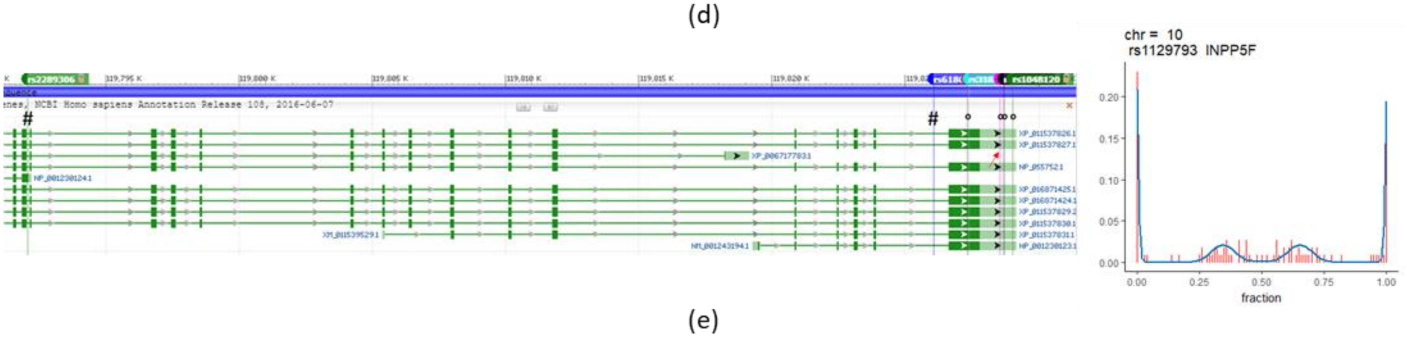

(e)

Supplementary Fig. 18 Isoform-specific maps and mixture distribution plots of (non-)significant SNPs located in the corresponding gene(s). (a) *PPIEL/BMP8A*: more complex patterns of imprinting are observed here which could be explained by overlapping transcripts. (b) *NAP1L5/HERC3*: an imprinted SNP located in *NAP1L5* and a non-imprinted one located in *HERC3*, indicating that only *NAP1L5* is imprinted. (c) *GRB10*: clearly not imprinted. (d) *MEST/MESTIT1*: SNP that looks (partially) imprinted, but was missed due to technical issues. (e) *INPP5F*: clearly not imprinted. On the transcript map SNPs that were filtered for technical reasons are marked with #, filtered imprinted SNPs that could be imprinted with *, non-imprinted SNP with ° and imprinted SNPs with "i". The SNP corresponding to the mixture distribution is indicated with a red arrow.

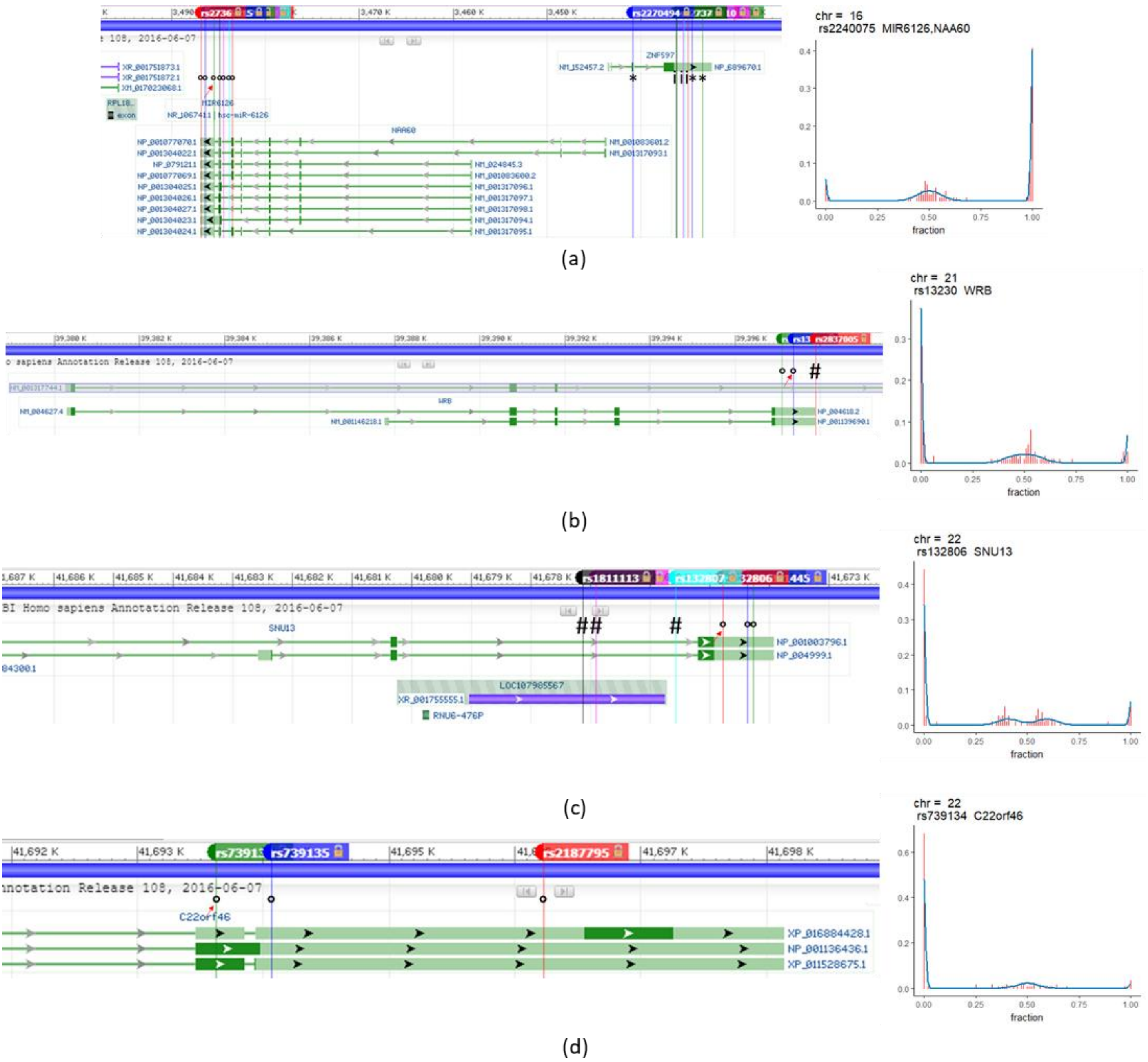

Supplementary Fig. 19 Isoform-specific maps and mixture distribution plots of (non-)significant SNPs located in the corresponding gene(s). (a) *ZNF597/NAA60/MIR6126*: the transcript map shows that indeed only *ZNF597* is imprinted. (b) *WRB*: clearly not imprinted. (c) *SNU13*: clearly not imprinted. (d) *C22orf46*: clearly not imprinted. On the transcript map SNPs that were filtered for technical reasons are marked with #, filtered imprinted SNPs that could be imprinted with *, non-imprinted SNP with ° and imprinted SNPs with “i”. The SNP corresponding to the mixture distribution is indicated with a red arrow.

e. Validation of imprinted pseudogenes

In the set of imprinted genes, some pseudogenes were detected: *CPHL1P*, *MTCO1P12*, *MTRNR2L1*, *USP32P2* and *ZNF300P1*. To verify that the pseudogene is imprinted rather than the original gene, additional validation was required. Reads containing the putatively imprinted SNPs were extracted from the BAM files. Next, a consensus sequence for each gene was constructed with multiple sequence alignment. Similar nucleotide sequences were then detected with BLAST (https://blast.ncbi.nlm.nih.gov/) against the human genome (blastn, “Human genomic + transcript”). For *CPHL1P*, *ZNF300P1* and *USP32P2* we indeed found high similarity with the pseudogenes and not with the original gene. However, for *MTCO1P12* and *MTRNR2L1* evidence was contradictory: though

aligning to both pseudogenes and original mitochondrial genes (even better to the latter for *MTRNR2L1*), imprinting of pseudogenes rather than mitochondrial artefacts is supported by (i) the observation of heterozygous samples (partial imprinting) for both genes (*MTCO1P12*, Supplementary Fig. 1(a); *MTRNR2L1*, Supplementary Fig. 9(e)-(h)) which is incompatible with mitochondrial genes; (ii) the perfect match between observed alleles and pseudogene dbSNP alleles; (iii) varying allele fractions for *MTRNR2L1* between SNPs (Supplementary Fig. 9(e)-(h)), incompatible with mitochondrial inheritance. Nevertheless, as the possibility of mitochondrial artefacts cannot be excluded, these loci are designated as candidate imprinted.

Supplementary Note 4. Differential imprinting

a. Differentially imprinted SNPs per subtype

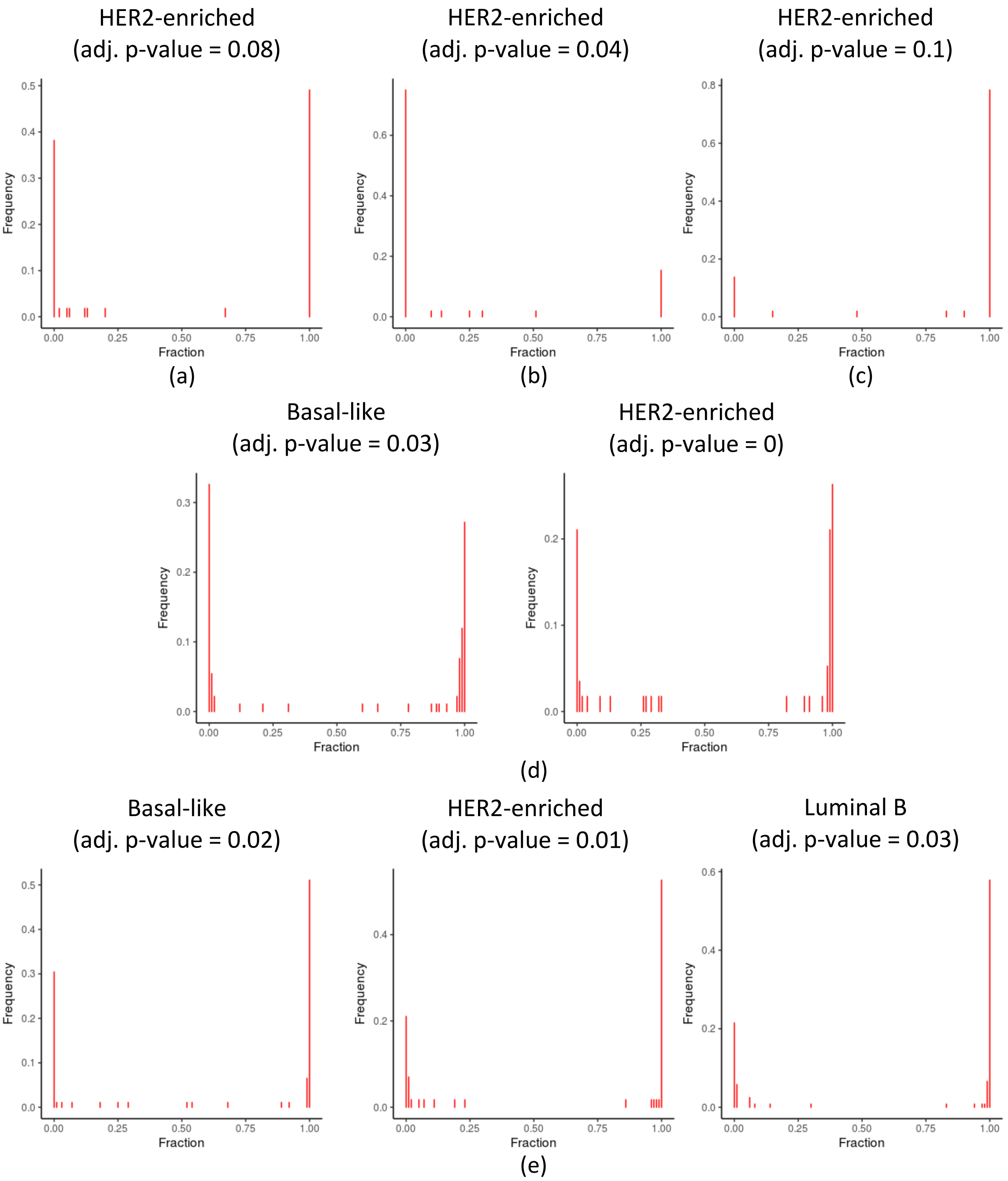

Supplementary Fig. 20 Mixture distributions of SNP positions with differential imprinting between control samples and cancer subtypes. The significant subtypes are here shown. (a) rs1053900 (*MEG3*) (b) rs4378559 (*MEG3*) (c) rs12890215 (*MEG3*) (d) rs3741219 (*H19*) (e) rs2839703 (*H19*)

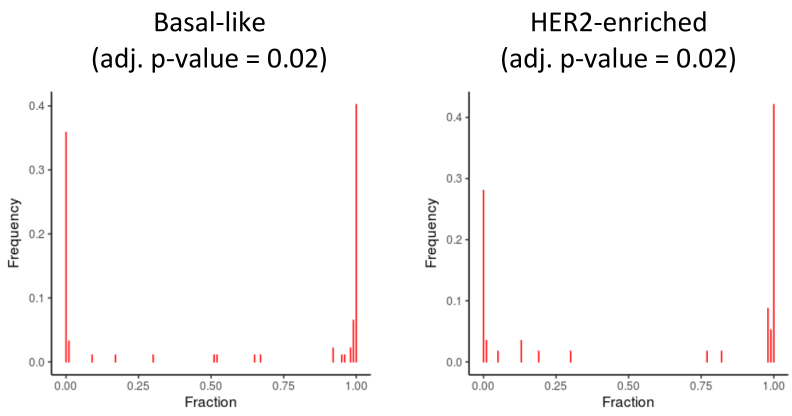

(a)

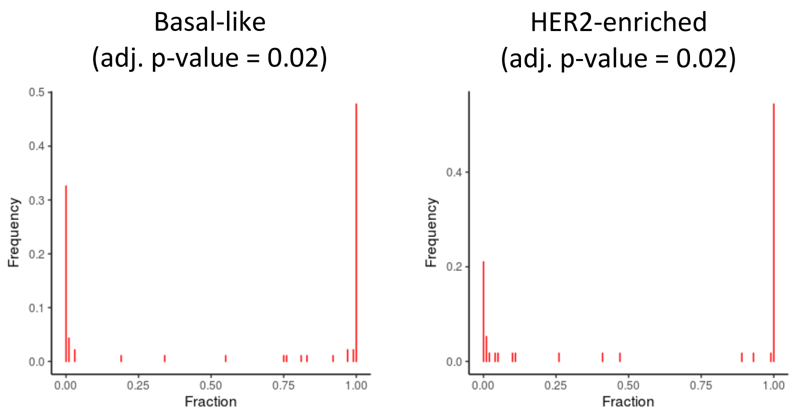

(b)

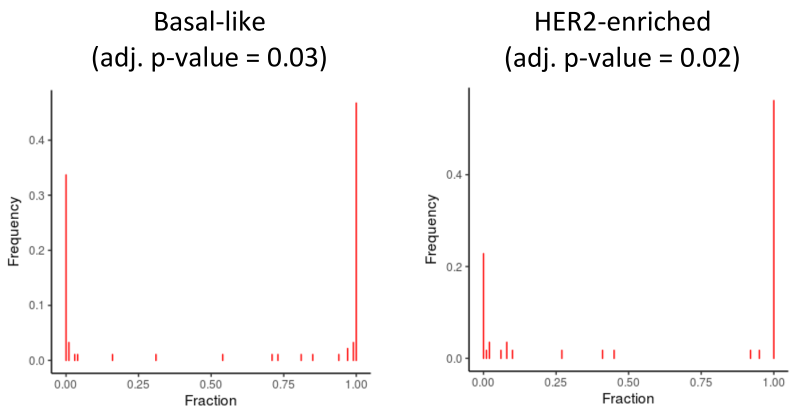

(c)

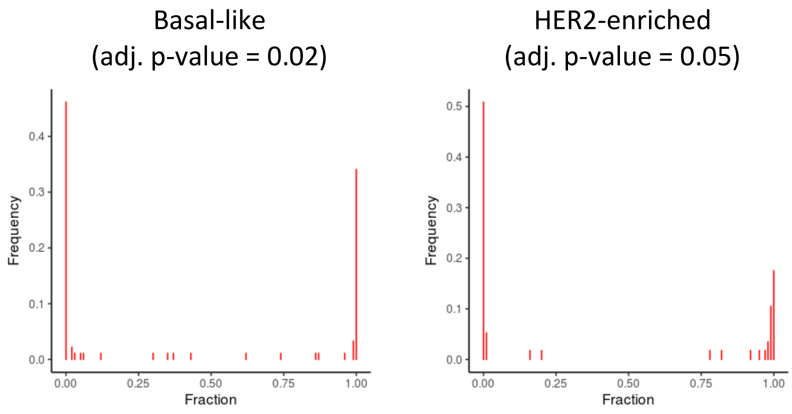

(d)

Supplementary Fig. 21 Mixture distributions of SNP positions with differential imprinting between control samples and cancer subtypes. The significant subtypes are here shown. (a) rs10840159 (*H19*) (b) rs2839702 (*H19*) (c) rs2839701 (*H19*) (d) rs2067051 (*H19*)

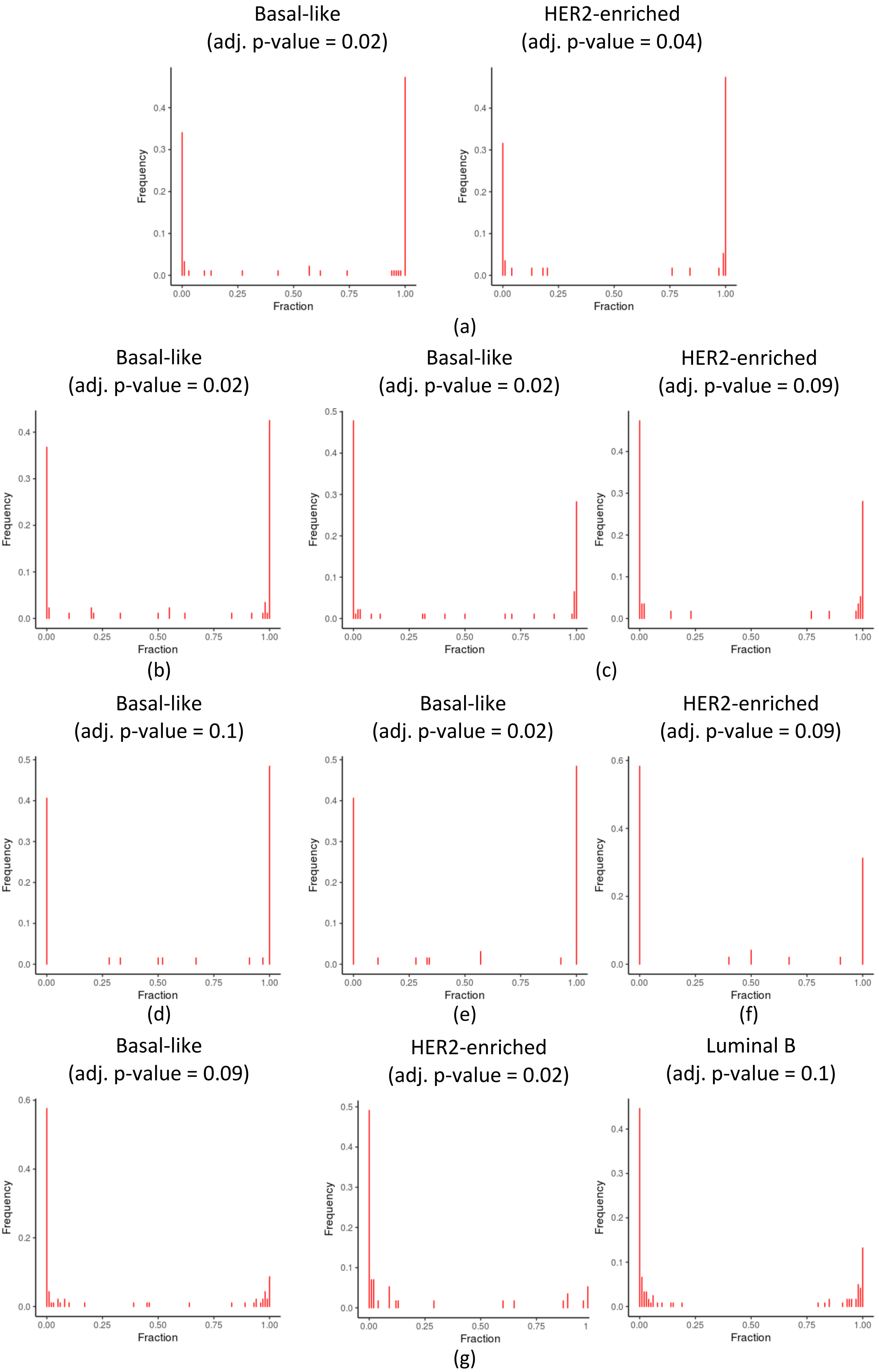

Supplementary Fig. 22 Mixture distributions of SNP positions with differential imprinting between control samples and cancer subtypes. The significant subtypes are here shown. (a) rs2075745 (*H19*) (b) rs2075744 (*H19*) (c) rs2839698 (*H19*) (d) rs7582864 (*ZDBF2*) (e) rs3732084 (*ZDBF2*) (f) rs1975597 (*ZDBF2*) (g) rs2585 (*IGF2*)

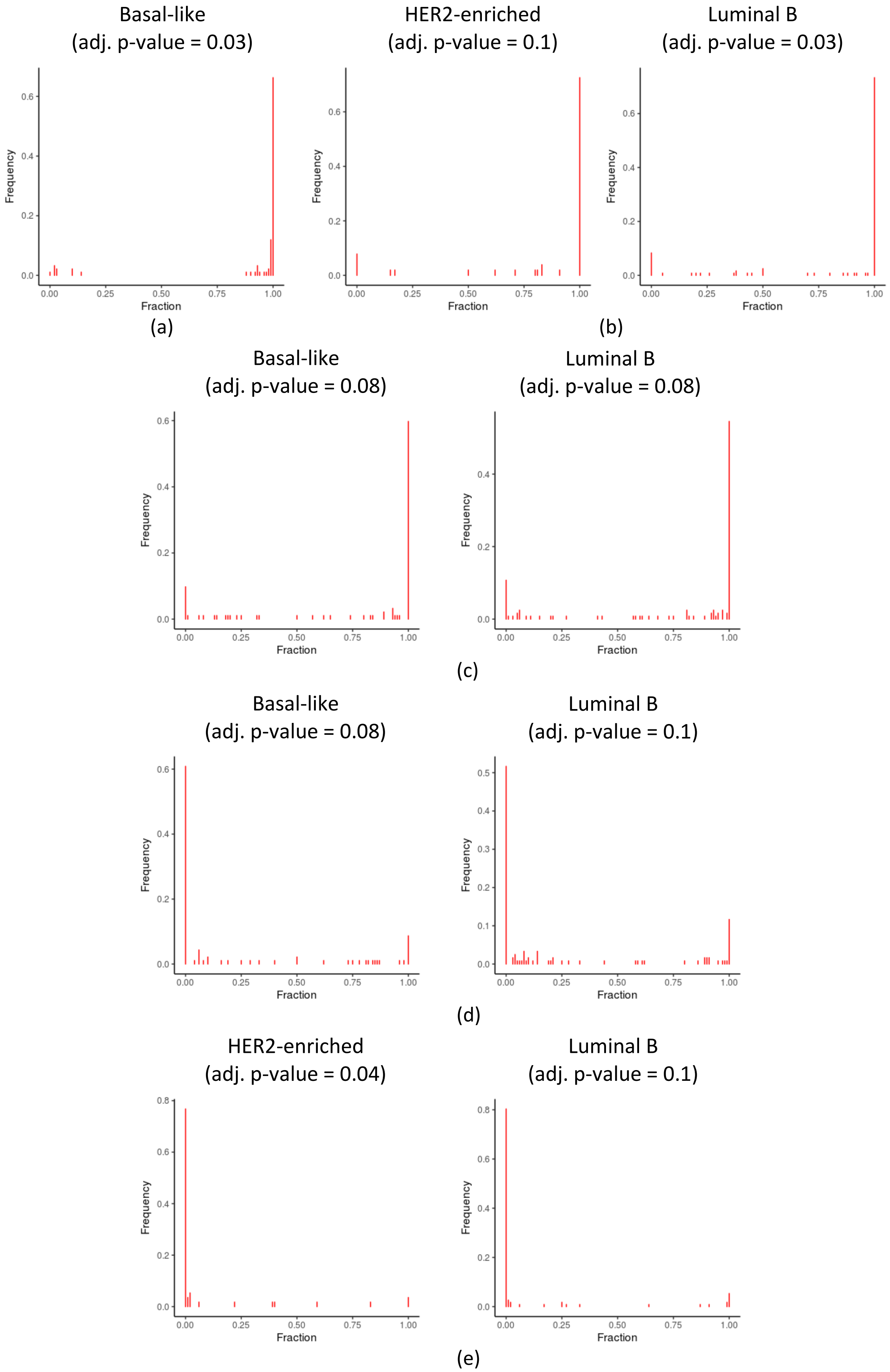

Supplementary Fig. 23 Mixture distributions of SNP positions with differential imprinting between control samples and cancer subtypes. The significant subtypes are here shown. (a) rs7873 (*IGF2*) (b) rs6059869 (*HM13*) (c) rs8110350 (*ZNF331*) (d) rs8110538 (*ZNF331*) (e) rs7810469 (*PEG10*)

b. *Biallelically expressing sample identification by RNA genotyping*

Validation of heterozygosity of biallelically expressing samples in WES data was limited due to low coverage, hence identification of such samples was also performed using RNA-seq data. To avoid the introduction of bias by imposing allelic-ratio based cut-offs, genotypes were called in all samples for the DI SNPs with SeqEM. To avoid that samples with low fractions of the silenced allele were automatically called homozygous, the HWE option of SeqEM was used. The estimated number of heterozygous samples (based on HWE, $2P_AP_T$*total#samples) was used to provide an estimate of the total fraction of biallelically expressing samples (Supplementary Table 5).

Supplementary Table 5 Number of observed and expected heterozygous samples in RNA-seq data for SNPs with DI for control, tumour and different breast cancer subtypes. Genotyping was done with SeqEM assuming Hardy Weinberg equilibrium to estimate the observed number of heterozygous samples. The expected number, on the other hand, is calculated by HWE as $2P_AP_T$*total#samples.*

SNP	Gene	Observed/Expected Heterozygous Samples						% Observed/Expected Heterozygous Samples					
		Control	Tumour	LumA	LumB	HER2	BL	Control	Tumour	LumA	LumB	HER2	BL
rs1053900	*MEG3*	0/56	11/245	2/112	3/59	**5/27**	1/43	0	4.5	1.8	5.1	**18.3**	2.3
rs4378559	*MEG3*	0/37	27/147	8/69	10/34	**5/17**	4/24	0	18.4	11.6	29.7	**29.5**	16.3
rs12890215	*MEG3*	1/38	30/150	7/71	13/34	**4/17**	6/25	2.6	20	9.9	38.1	**23.4**	23.6
rs10863	*MEST*	8/31	**101/137**	**41/62**	**36/33**	**16/15**	7/25	26.1	**73.6**	**66.5**	**109.5**	**103.3**	28
rs3741219	*H19*	0/56	15/251	2/113	1/60	**6/28**	**6/46**	0	6	1.8	1.7	**21.3**	**13.2**
rs2839704	*H19*	0/53	**14/239**	1/107	**3/57**	**4/27**	**6/43**	0	**5.9**	0.9	**5.3**	**14.9**	**13.8**
rs2839703	*H19*	0/53	**13/239**	1/107	**3/57**	**3/27**	**6/43**	0	**5.4**	0.9	**5.3**	**11.2**	**13.8**
rs10840159	*H19*	0/56	16/251	1/113	3/60	**6/28**	**6/46**	0	6.4	0.9	5.0	**21.3**	**13.2**
rs2839702	*H19*	0/56	16/251	2/113	4/60	**3/28**	**7/46**	0	6.4	1.8	6.7	**10.6**	**15.4**
rs2839701	*H19*	0/56	16/251	2/113	4/60	**3/28**	**7/46**	0	6.4	1.8	6.7	**10.6**	**15.4**
rs2067051	*H19*	1/56	16/252	0/114	2/60	**4/28**	**10/45**	1.8	6.4	0	3.3	**14.1**	**22**
rs2075745	*H19*	1/56	16/252	2/114	2/60	**5/28**	**7/45**	1.8	6.4	1.8	3.3	**17.6**	**15.4**
rs2075744	*H19*	1/50	18/243	2/109	4/58	2/28	**10/43**	2	7.4	1.8	6.9	7.1	**23**
rs2839698	*H19*	0/56	14/251	0/113	3/60	**4/28**	**7/46**	0	5.6	0	5	**14.1**	**15.3**
rs7582864	*ZDBF2*	5/44	58/145	30/72	11/33	5/11	**12/28**	11.3	40	41.6	33.8	45.5	**42.6**
rs3732084	*ZDBF2*	5/47	48/162	23/80	7/35	7/16	**11/28**	10.6	29.7	28.7	19.7	44.4	**39.2**
rs1975597	*ZDBF2*	1/48	69/182	25/84	22/42	**13/21**	8/33	2.1	37.9	29.9	52.3	**62.4**	24.6
rs2585	*IGF2*	0/46	18/205	4/93	**5/49**	**3/23**	**6/37**	0	8.8	4.3	**10.2**	**13**	**16.1**
rs7873	*IGF2*	0/21	5/96	0/43	1/23	3/11	**1/17**	0	5.2	0	4.4	28.2	**5.7**
rs6059869	*HM13*	8/34	66/150	25/68	**16/37**	**10/16**	15/27	23.7	44	36.6	**43.8**	**64.4**	55.3
rs6059873	*HM13*	4/33	**42/147**	10/67	**13/36**	**6/16**	**12/27**	12.2	**28.6**	15	**36.6**	**37.9**	**44.9**
rs8110538	*ZNF331*	4/50	49/222	11/100	**18/53**	6/25	**13/40**	8	22.1	11	**33.8**	24.4	**32.1**
rs8110350	*ZNF331*	6/48	53/216	14/97	**15/51**	8/24	**15/39**	12.4	24.5	14.4	**29.2**	32.8	**38.1**
rs7810469	*PEG10*	1/22	22/97	5/44	**10/22**	**5/11**	1/18	4.5	22.7	11.4	**45**	**45**	5.5

*Significant DI SNPs per subtype are shown in bold.

c. *Degree of imprinting linked to survival and age*

It was evaluated whether DI was associated with survival in tumour samples. Based on a Cox proportional hazard model (see Methods, Section 5), adjusting for age, and considering allelic ratio (AR = allele count least expressed allele/allele count most expressed allele) as measure for degree of imprinting, the *ZDBF2* AR was significantly associated with poorer survival (for two of the three SNPs with significant DI in ZDBF2; FDR-adjusted p = 0.027 and 0.086 for rs3732084 and rs1975597, respectively). Also when survival analysis was performed solely on the putative heterozygous samples (see Supplementary Methods), *ZDBF2* AR was associated with significant lower survival. The degree of imprinting (AR) was not correlated to age for tumour or control data (Supplementary Table 6).

The logCPM-value of the least expressed allele count was also linked to survival using an age adjusted Cox proportional hazard model. However, here too little events occurred to accurately draw

conclusion from this analysis. Both the allelic ratio as well as the logCPM-value of the least expressed allele count were also correlated to age (Pearson's correlation test), but no significant correlations were found (Supplementary Table 6). The same conclusions were obtained when only taking the putative heterozygous samples into account.

Supplementary Table 6 Correlation of imprinting degree with age. Pearson's *r* (*r*) and FDR-adjusted p-values (p) of tumour and control subsets are shown for all DI SNPs. The degree of imprinting was once implemented as the allelic ratio (AR) and once as the normalised count of the least expressed allele (logCPM).

		AR				logCPM			
SNP	Gene	Tumour		Control		Tumour		Control	
		r	p	*r*	p	*r*	p	*r*	p
rs3741219	*H19*	-0.02	0.79	-0.04	0.77	-0.09	0.29	0.11	0.69
rs2839704	*H19*	-0.03	0.79	-0.06	0.72	-0.04	0.57	0.14	0.69
rs2839703	*H19*	-0.02	0.79	-0.12	0.62	0	0.94	0.09	0.69
rs10840159	*H19*	-0.02	0.79	-0.07	0.72	-0.10	0.28	0.14	0.69
rs2839702	*H19*	-0.04	0.68	0.02	0.87	-0.10	0.28	0.10	0.69
rs2839701	*H19*	-0.04	0.68	0.04	0.77	-0.04	0.57	0.06	0.71
rs2067051	*H19*	-0.05	0.68	-0.08	0.72	-0.06	0.57	-0.04	0.71
rs2075745	*H19*	-0.05	0.68	-0.06	0.72	-0.04	0.57	-0.05	0.71
rs2075744	*H19*	-0.07	0.68	-0.20	0.62	-0.04	0.57	-0.09	0.69
rs2839698	*H19*	-0.06	0.68	-0.15	0.62	-0.07	0.57	-0.06	0.71
rs2585	*IGF2*	0.02	0.79	0.07	0.72	-0.05	0.57	0.12	0.69
rs7873	*IGF2*	-0.04	0.68	0.11	0.62	-0.12	0.26	0.29	0.07
rs6059869	*HM13*	-0.06	0.68	0.04	0.77	-0.06	0.57	0.08	0.69
rs6059873	*HM13*	-0.07	0.68	0.08	0.72	-0.05	0.57	0.09	0.69
rs8110538	*ZNF331*	0	1	0.07	0.72	0.02	0.66	0.04	0.71
rs8110350	*ZNF331*	0.01	0.88	0.06	0.72	0.04	0.57	0.07	0.71
rs7810469	*PEG10*	0.01	0.93	-0.12	0.62	0.03	0.66	-0.08	0.70
rs1053900	*MEG3*	-0.05	0.68	-0.02	0.87	-0.03	0.66	0.04	0.71
rs4378559	*MEG3*	-0.04	0.68	0.12	0.62	-0.04	0.57	0.13	0.69
rs12890215	*MEG3*	-0.02	0.86	0.01	0.88	-0.04	0.57	0.03	0.79
rs10863	*MEST*	0.06	0.68	-0.16	0.62	0.04	0.57	-0.09	0.69
rs3732084	*ZDBF2*	0.03	0.79	0.13	0.62	0.05	0.57	0.14	0.69
rs1975597	*ZDBF2*	0.04	0.68	-0.11	0.62	0.01	0.81	-0.11	0.69

Supplementary Note 5. Differential expression

Significant DE was found in 92% of the imprinted SNPs (111 out of 121) for all tumour samples. In the different subtypes 90% (109 SNPs), 89% (108 SNPs), 89% (108 SNPs) and 92% (111 SNPs) showed significant DE for HER2, BL, LumA and LumB respectively (Supplementary Table 7). In this set of differentially expressed SNPs most loci were downregulated. 87% (97 out of 111 SNPs) were detected with a negative log fold change for the tumour samples. 95% (104 out of 1109 SNPs), 86% (93 out of 108 SNPs), 93% (100 out of 108 SNPs) and 85% (94 out of 1141 SNPs) of the deregulated loci were downregulated for HER2, BL, LumA and LumB respectively (Supplementary Table 7). The FDR-adjusted p-values and log fold changes for all SNPs can be found in Supplementary Data 5.

Supplementary Table 7 Number of significant differentially expressed imprinted SNPs in control versus tumour data and control versus the different breast cancer subtypes. The number of SNPs with an FDR-adjusted p-value ≤ 0.05 are shown in column "# DE" with the percentage between brackets. The number and percentage of upregulated (positive log fold change) and downregulated (negative log fold change) SNPs are shown in the other columns.

Control vs	# DE (%)	# Up (%)	# Down (%)
Tumour	111 (92)	14 (13)	97 (87)
HER2	109 (90)	5 (5)	104 (95)
BL	108 (89)	15 (14)	93 (86)
LumA	108 (89)	8 (7)	100 (93)
LumB	111 (92)	17 (15)	94 (85)

Afterwards, we also examined differential expression for the detected imprinted genes by taking the sum of the CPM-values of the SNPs corresponding to an imprinted gene. Again this analysis was done for tumour samples as well as the different subtypes. FDR-adjusted p-values are shown in Supplementary Table 8. As two SNPs (rs1056905 and rs1800900) were located in the exonic region of two genes, these genes (*GLIPR1/KRR1* and *GNAS/GNAS-AS1*) were here analysed as one. Hence, in this analysis only 29 imprinted genes were studied. The results again confirm that imprinting is heavily deregulated in breast cancer and the different subtypes. We found 100% DE in the aggregated tumour data and 93% (27 out of 29 genes), 86% (25 out of 29 genes), 90% (26 out of 29 genes) and 97% (28 out of 29 genes) in HER2, BL, LumA and LumB respectively (Supplementary Table 9). In the tumour samples all genes were differentially expressed, 24 downregulated (76%) and 7 (24%) upregulated. Also in the varying subtypes most genes were differentially expressed. Only for 9 genes no DE was found in some of the subtypes, namely *H19*, *HM13* (borderline in HER2, significant in other subtypes), *LINC01139*, *LOC100294145*, *PEG10*, *PTX3*, *USP32P2* (borderline in BL, significant in other subtypes) and *ZNF597*. Again, most genes were downregulated with around 26 downregulated genes for all subtypes.

Supplementary Table 8 FDR-adjusted p-values (p) and log fold changes (logFC) of differential expression analysis. CPM-values of the corresponding SNPs per gene were used to compare control versus diseased samples. Differential expression between control and tumour was analysed with a Wilcoxon Rank Sum test, while analysis per subtype was done with a Kruskal-Wallis test and Dunn's post-hoc test.*

Gene	Tumour		HER2		BL		LumA		LumB	
	logFC	p	logFC	p	logFC	p	logFC	p	logFC	p
BCR	-0.83	**4.07E-06**	-0.88	**1.24E-03**	-0.72	**2.92E-05**	-0.96	**2.35E-05**	-0.66	**3.70E-04**
DLK1	1.15	**9.54E-31**	2.82	**6.02E-10**	2.46	**5.72E-19**	-4.16	**4.01E-25**	0.61	**1.46E-25**
GNAS-AS1/GNAS	-2.06	**9.33E-19**	-1.70	**3.92E-08**	-0.86	**2.64E-04**	-3.83	**5.51E-19**	-2.01	**5.03E-19**
H19	-0.75	**4.27E-02**	-1.09	**4.44E-03**	-0.67	**7.12E-04**	-0.58	4.32E-01	-1.07	**2.17E-03**
HM13	0.47	**1.53E-05**	0.27	5.43E-02	0.59	**2.38E-05**	0.37	**9.09E-04**	0.64	**1.05E-06**
HOTAIRM1	-1.79	**5.43E-26**	-2.46	**1.08E-17**	-0.59	**3.88E-06**	-2.13	**2.28E-22**	-2.57	**5.66E-24**
IGF2	-1.15	**2.10E-33**	-2.37	**2.22E-22**	0.14	**3.50E-36**	-1.38	**2.59E-15**	-2.31	**7.52E-29**
LINC01139	0.20	**7.32E-04**	-0.49	**1.23E-03**	1.27	1.66E-01	-0.34	**2.58E-04**	0.16	**5.37E-03**
LOC100294145	0.58	**9.12E-04**	0.76	**2.60E-04**	1.01	**6.98E-06**	0.13	2.51E-01	0.84	**4.30E-06**
MEG3	-2.41	**1.08E-45**	-2.15	**1.87E-16**	-3.06	**3.13E-41**	-2.16	**1.67E-29**	-2.73	**1.06E-36**
MEST	-1.59	**1.23E-31**	-1.68	**3.23E-13**	-0.94	**6.47E-15**	-1.86	**6.30E-31**	-1.74	**2.53E-19**
MTCO1P12	0.14	**4.09E-04**	-0.53	**3.86E-02**	0.86	**2.10E-04**	0.17	**2.17E-03**	-0.47	**9.45E-04**
MTRNR2L1	-1.56	**1.84E-27**	-1.16	**2.25E-07**	-1.58	**2.43E-16**	-1.61	**2.53E-23**	-1.85	**4.93E-24**
NAP1L5	-1.13	**1.60E-33**	-1.58	**1.06E-24**	-1.15	**1.22E-21**	-1.00	**4.60E-22**	-1.25	**2.88E-25**
PAX8-AS1	-0.60	**3.00E-06**	-0.83	**9.19E-04**	-0.38	**1.67E-04**	-0.56	**9.74E-05**	-0.74	**1.46E-05**
PEG10	0.77	**1.32E-03**	0.85	5.51E-02	0.87	2.96E-01	0.78	**5.59E-05**	0.70	**3.56E-04**
PEG3	0.01	**1.79E-21**	-0.70	**3.82E-12**	0.74	**6.52E-12**	-0.40	**5.64E-17**	0.28	**6.85E-16**
PLAGL1	-2.54	**3.03E-41**	-3.02	**1.98E-21**	-1.34	**1.19E-08**	-2.84	**1.25E-35**	-3.61	**3.12E-42**
*PLIN1***	-5.53	**1.31E-42**	-5.95	**1.11E-21**	-7.24	**3.36E-36**	-5.32	**5.96E-30**	-5.17	**5.17E-26**
*PTX3***	-1.45	**5.53E-25**	-3.83	**1.68E-19**	0.11	**1.30E-01**	-1.62	**8.37E-22**	-4.47	**2.57E-33**
PWAR6	-1.33	**1.41E-32**	-1.73	**5.23E-19**	-2.22	**6.45E-35**	-1.15	**3.00E-19**	-1.03	**7.94E-20**
*RP11-109L13.1***	-1.75	**9.62E-07**	-1.20	**3.09E-02**	-1.80	**2.38E-05**	-1.68	**3.24E-06**	-2.20	**1.48E-05**
SNHG14	-1.20	**9.54E-31**	-1.19	**5.90E-14**	-1.90	**4.27E-32**	-1.16	**1.22E-21**	-0.88	**1.87E-16**
SNRPN	-0.55	**1.20E-13**	-0.57	**4.51E-07**	-1.22	**1.06E-22**	-0.42	**2.25E-07**	-0.41	**2.59E-07**
*USP32P2***	-0.57	**6.38E-04**	-1.12	**2.09E-03**	-0.20	5.51E-02	-0.46	**1.18E-03**	-0.93	**4.12E-04**
ZDBF2	-1.34	**2.49E-33**	-2.12	**4.93E-24**	-1.28	**1.14E-18**	-1.28	**2.81E-24**	-1.25	**5.11E-23**
ZNF300P1	-2.87	**1.32E-36**	-2.45	**3.90E-14**	-3.49	**3.67E-27**	-2.75	**6.73E-28**	-3.11	**2.62E-26**
ZNF331	-0.91	**2.76E-28**	-1.00	**1.36E-18**	-1.02	**4.04E-18**	-0.93	**4.60E-22**	-0.75	**3.01E-16**
ZNF597	-0.09	**1.68E-02**	-0.13	**3.88E-02**	-0.84	**6.04E-12**	0.07	2.79E-01	0.08	4.01E-01

*Significant DE is shown in bold. **Candidate imprinted genes.

Supplementary Table 9 Number of significant differentially expressed imprinted genes in control versus tumour data and control versus the different breast cancer subtypes. The number of genes with an FDR-adjusted p-value ≤ 0.05 are shown in column "# DE" with the percentage between brackets. The number and percentage of upregulated (positive log fold change) and downregulated (negative log fold change) genes are shown in the other columns.

Control vs	# DE (%)	# Up (%)	# Down (%)
Tumour	29 (100)	7 (24)	22 (76)
HER2	27 (93)	2 (7)	25 (93)
BL	25 (86)	6 (24)	19 (76)
LumA	26 (90)	3 (12)	23 (88)
LumB	28 (97)	6 (21)	22 (79)

Verification of DI associated DE

Whole exome sequencing data were used to verify the impact of DI on differential expression. Biallelically expressing samples were here conservatively defined as samples with a heterozygous WES-based genotype and an AR of 0.2 or more (cf. Supplementary Fig. 14). Not-biallelically expressing heterozygotes, conversely, were samples with a heterozygous WES genotype and an AR of 0.2 or less. A raw count threshold of 6 per SNP was used to limit loss of data. The combination of genotyping data and an AR-threshold was used to make the identification of biallelically expressing samples less sensitive to sequencing errors. When no biallelically expressing heterozygotes were found amongst the control samples, we assumed that biallelic expression did not occur in control tissue and all control samples were considered to be monoallelically expressing. Expression values were the same as the ones used for the SNP subtype analysis. The data were thus split into biallelically expressing tumour, monoallelically expressing tumour, biallelically expressing control and monoallelically expressing control samples (Supplementary Table 10). Overall differential expression between all subgroups was tested with a Kruskal-Wallis test. When FDR-corrected Kruskal-Wallis p-values were smaller than 0.1, enough evidence for differential expression among subgroups was assumed to further explore the changes between the subgroups with a Dunn's test. Dunn's test p-values were not further FDR-corrected as the remaining number of tests performed was very small (Supplementary Table 11).

Supplementary Table 10 Number of biallelically expressing (BA) and monoallelically expressing (MA) samples in tumour and control samples based on the allelic ratio and whole exome sequencing genotype. Mean CPM-values per subgroup and their confidence interval are also shown.

Gene	SNP	# BA tumour	# MA tumour	# BA control	# MA control	CPM BA tumour	CPM MA tumour	CPM BA control	CPM MA control
ZNF331	rs8109631	46	126	3	30	0.23[0.14-0.39]	0.32 [0.24-0.49]	0.31 [0.26-0.53]	0.70 [0.54-0.83]
HM13	rs1115713	17	63	3	13	0.30 [0.25-0.56]	0.17 [0.12-0.28]	0.29 [0.24-0.32]	0.17 [0.14-0.18]
*USP32P2**	rs141915702	9	6	5	0	0.12 [0.11-0.19]	0.18 [0.15-0.31]	0.38 [0.22-0.39]	NA
ZDBF2	rs3732084	6	51	0	83**	0.15 [0.07-0.22]	0.09 [0.07-0.12]	NA	0.12 [0.07-0.18]
H19	rs2839701	5	46	0	83**	2.72 [2.08-3.16]	5.15 [2.60-7.55]	NA	4.31 [2.44-9.41]

*Candidate imprinted genes. **When no heterozygote BA samples were observed, all control samples were considered to be MA

Supplementary Table 11 Differential expression analysis between biallelically expressing (BA) and monoallelically expressing (MA) samples in control and tumour tissue. Differential expression between the subgroups with a Dunn's test was only tested when the FDR-adjusted p-value of the Kruskal-Wallis test was significant with a significance threshold of 0.1.*

Gene	SNP	p Kruskal-Wallis	p BA tumour vs MA control	p BA tumor vs MA tumor	p BA control vs MA control	p BA control vs BA tumour	p MA control vs MA tumour
ZNF331	rs8109631	**3.287E-09**	**9.089E-12**	**0.003544**	0.05928	0.1445	**2.133E-08**
HM13	rs1115713	**0.003314**	**0.0003734**	**0.0001473**	**0.06941**	0.3192	0.2032
*USP32P2***	rs141915702	0.1215	NA	NA	NA	NA	NA
ZDBF2	rs3732084	0.1896	NA	NA	NA	NA	NA
H19	rs2839701	0.2658	NA	NA	NA	NA	NA

*Significant results are shown in bold. **Candidate imprinted genes.

Supplementary Note 6. DE and DI linked to copy number variation

LogCPM-values of all genes (counts obtained from firebrowse.org and normalised with EdgeR) were compared with CNV data with a linear model in tumour samples. A second linear model was constructed including the breast cancer subtypes. For 17 of the 23 imprinted genes a significant link between expression and CNV was found (Supplementary Table 12). For each gene, a contingency table was made comparing CNV with DE. A sample was called up- or downregulated when featured by a normalised logCPM-value outside of the 95% confidence interval of the control data (Supplementary Data 6). Results are graphically summarised in Supplementary Fig. 24.

Supplementary Table 12 Degrees of freedom (df), F-values (F) and FDR-adjusted p-values (p) for imprinted genes of linear models for expression (dependent variable) and copy number variation (model 1; 2 degrees of freedom) and also expression, copy number variations and breast cancer subtype (model 2; 2 and 4 degrees of freedom respectively).*

Gene	CNV (model 1)			CNV (model 2)			Subtypes (model 2)		
	df	F	p	df	F	p	df	F	p
LINC01139	464	1.39	2.74E-01	460	1.41	2.69E-01	460	2.43	5.79E-02
PLAGL1	464	7.10	**1.76E-03**	460	10.08	**1.19E-04**	460	49.75	**9.95E-34**
SNRPN	464	65.38	**7.42E-25**	460	71.72	**5.92E-27**	460	2.26	**5.09E-09**
PEG10	464	3.80	**3.19E-02**	460	3.91	**2.80E-02**	460	4.35	**2.82E-03**
ZNF300P1	464	4.54	**1.71E-02**	460	4.91	**1.19E-02**	460	10.47	**8.37E-08**
PTX3	464	5.93	**4.70E-03**	460	9.54	**1.83E-04**	460	71.52	**1.02E-45**
PAX8-AS1	464	0.10	9.09E-01	460	0.10	9.09E-01	460	1.06	3.93E-01
PEG3	464	7.23	**1.69E-03**	460	7.21	**1.36E-03**	460	0.62	6.49E-01
DLK1	464	2.57	9.91E-02	460	2.68	8.92E-02	460	5.83	**2.47E-04**
MEST	464	3.98	**3.67E-06**	460	14.06	**3.40E-06**	460	1.69	1.66E-01
BCR	464	166.63	**6.63E-54**	460	182.50	**1.03E-57**	460	12.05	**6.52E-09**
GNAS	464	43.50	**2.22E-17**	460	49.86	**1.45E-19**	460	17.95	**4.78E-13**
PLIN1	464	6.82	**2.14E-03**	460	7.90	**7.52E-04**	460	19.39	**5.35E-14**
USP32P2	443	11.54	**3.32E-05**	439	11.69	**2.89E-05**	439	2.42	5.79E-02
NAP1L5	464	18.06	**9.23E-08**	460	18.71	**5.05E-08**	460	5.23	**6.57E-04**
ZNF331	464	48.73	**3.54E-19**	460	49.58	**1.45E-19**	460	3.02	**2.38E-02**
H19	465	0.11	9.09E-01	460	0.12	9.09E-01	460	0.35	**3.18E-06**
MEG3	464	0.19	1.37E-01	460	2.39	1.12E-01	460	11.55	**1.39E-08**
IGF2	463	3.78	**3.19E-02**	459	4.53	**1.62E-02**	459	24.06	**3.36E-17**
ZNF597	463	126.32	**1.92E-43**	459	138.86	**7.94E-47**	459	12.50	**3.83E-09**
GNAS-AS1	464	2.11	1.41E-01	460	2.13	1.38E-01	460	2.26	7.06E-02
HM13	464	34.05	**6.11E-14**	460	37.88	**2.26E-15**	460	14.05	**3.04E-10**
ZDBF2	464	7.92	**9.55E-04**	460	8.07	**6.90E-04**	460	3.18	**1.96E-02**

*Significant p-values are shown in bold.

For HM13, overexpression in tumour samples was compared to CNV data to ascertain that LOI was not associated with gain of the locus (given the observed re-expression of the previously silenced allele, DI here corresponds to LOI). Genotypes of the SNPs were called with SeqEM (assuming HWE) on the RNA-seq data to identify heterozygous, and thus LOI, samples. CNV data was downloaded from TCGA, a segment mean < -0.2 was called a loss, > 0.2 a gain, and in between neutral[6]. A χ^2 test compared LOI status with CNV (notLOI-gain vs notLOI-neutral/loss vs LOI-gain vs LOI-neutral/loss). No significant p-values were obtained showing that LOI occurs independently from CNV for HM13 (Supplementary Table 13).

Supplementary Table 13 Contingency table for LOI vs CNV of each imprinted HM13 SNP in tumour data. The p-values of the χ^2 test (p) comparing notLOI-gain vs notLOI-neutral/loss vs LOI-gain vs LOI-neutral/loss is also denoted in the table.

Position	30127392		30128553		30129046		30129099		30135990	
	LOI	nLOI	LOI	nLOI	LOI	nLOI	LOI	nLOI	LOI	nLOI
Gain	14	124	20	115	13	125	13	128	11	128
Neutral	25	282	40	268	24	290	28	284	18	297
Loss	0	9	3	7	1	9	0	10	0	10
p	0.55		0.83		0.67		1		0.45	

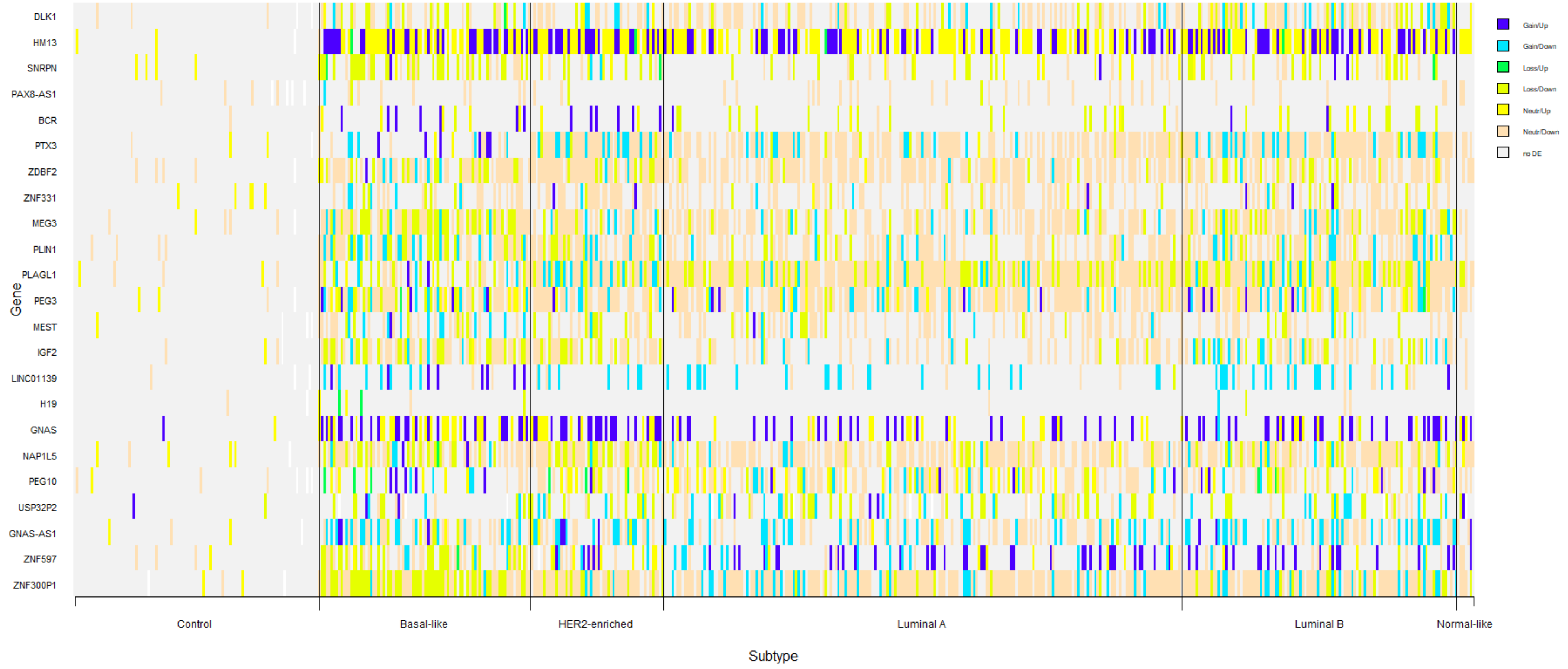

Supplementary Fig. 24 Overview of copy number variation and differential expression of the imprinted loci. For each SNP and all samples, the colour scale indicates significant DE and/or CNV. A samples is called up- or downregulated when the normalised logCPM-value outside of the 95%-confidence interval of control data. A loss was defined as a segment with mean < -0.2, a gain when it is > 0.2.

Supplementary Note 7. Preliminary gene network analysis

As many of the imprinted genes were downregulated in breast tumour data, we hypothesise that an imprinted gene network may be present in breast. The counts of all genes from the breast RNA-seq expression count file (obtained from firebrowse.org) were normalised with EdgeR and log-transformed to obtain logCPM-values. Differential expression analysis between tumour and control data was subsequently done with a Wilcoxon Rank Sum test. Spearman correlation was performed for all DE loci, yet as differential expression between tumours and controls was prominent and would thus affect correlation measures, we only considered tumour data. Many positive correlations were found between the downregulated genes and also the upregulated (*HM13* and *GNAS*) genes were positively correlated (Supplementary Fig. 25). DE genes that showed downregulation in cancer were also used for PCA (tumour data only) to evaluate potential co-expression. Also here, the first principal component suggests concerted action, given that virtually all loadings are negative, compatible with imprinted gene networks. Moreover, these analyses put forward *PLAGL1*, *MEG3* and *ZNF300P1* as candidate regulators, of which at least *MEG3* deregulation is independent of CNV but possibly methylation driven (cf. Results, Supplementary Fig. 26). Nevertheless, advanced co-expression analysis is required (also taking into account the impact of CNV and aberrant DNA methylation) to formally assess whether an imprinted gene network is indeed present and responsible for imprinted gene downregulation in breast cancer.

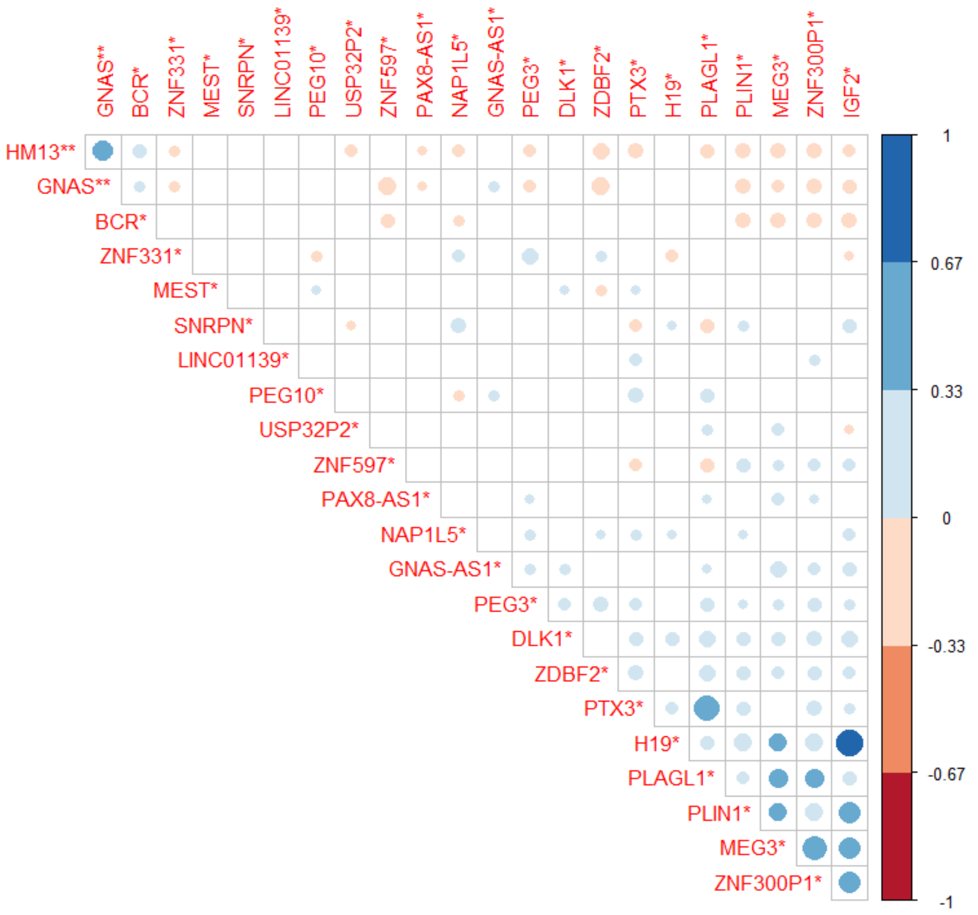

Supplementary Fig. 25 Correlation plot of imprinted genes in tumour data. Spearman correlation was used on the normalised expression counts. Significant correlations are indicated with a circle of which the colour, colour intensity and size are proportional to the correlation coefficient. Significantly downregulated genes are denoted with * and upregulated ones with **.

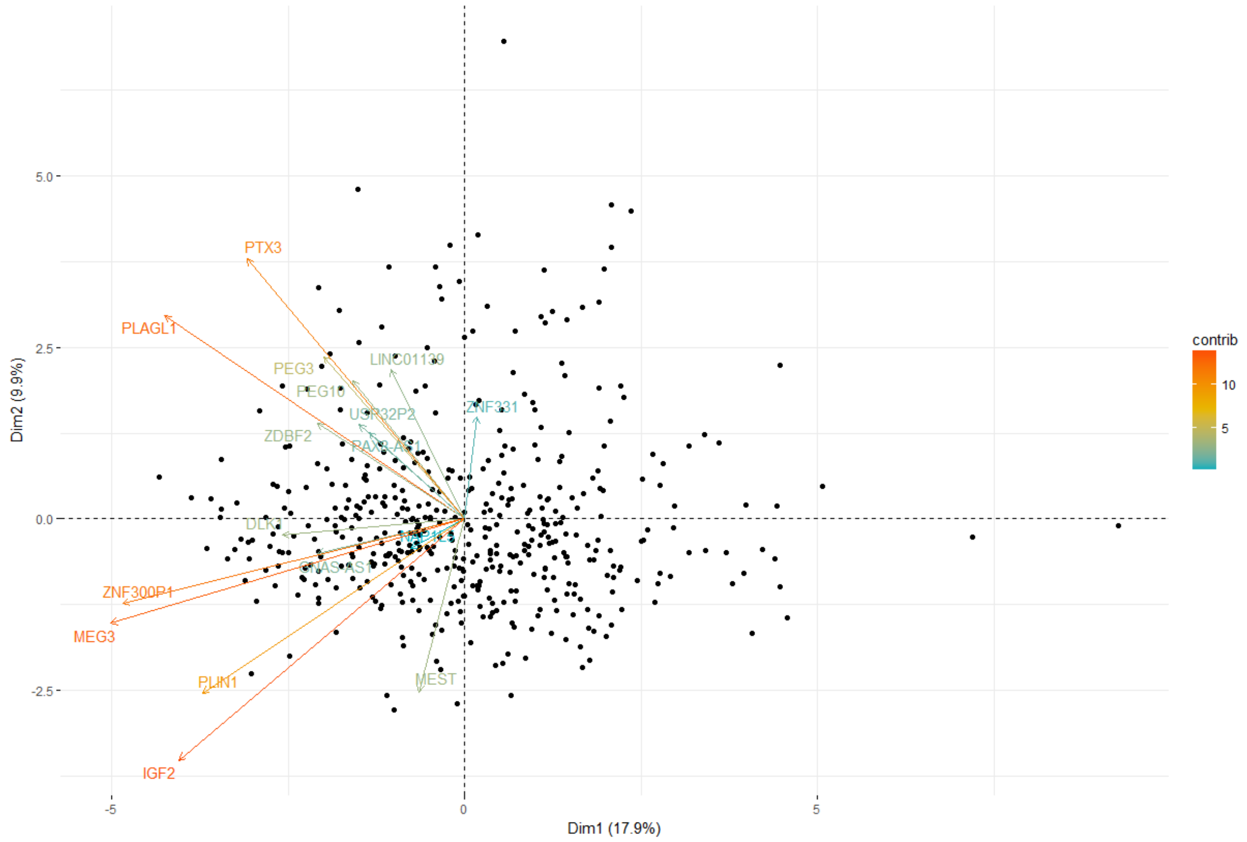

Supplementary Fig. 26 Principal component analysis biplot of the downregulated genes in tumour data. The arrows and their colour indicate the influence of each gene on the principal components (PC). The first PC accounts for 17.9% of the variance and the second for 9.9%.

Supplementary Note 8. Infiltrating lymphocytes

We evaluated lymphocyte infiltration in tumour tissue as possible explanation of DI of *MEST* and *HM13*, given prior evidence for biallelic *MEST* expression (isoform 2) in blood, and the relevant results for *HM13* in breast cancer in this study[7].

No significant correlation (p-value of 0.9 and R of 0.0006 with a correlation permutation test if only the $2P_AP_T$ highest fraction is taken into account) was found between the percentage of infiltrating lymphocytes and biallelic expression of *MEST* (estimated as the allelic ratio). Furthermore, *MEST* was downregulated in tumour samples, decreasing the probability that infiltrating lymphocytes may be relevant in *MEST* DI.

Only for the last *HM13* SNP (rs1115713), a significant correlation between the percentage of infiltrating lymphocytes and biallelic expression was found (p-value of 0.01 and R of 0.15), though here no DI was detected. Note that this SNP is exonic in *MCTS2P* and the correlation could be hence rather due to biallelic *MCTS2P* expression than *HM13* expression.

Supplementary Figures

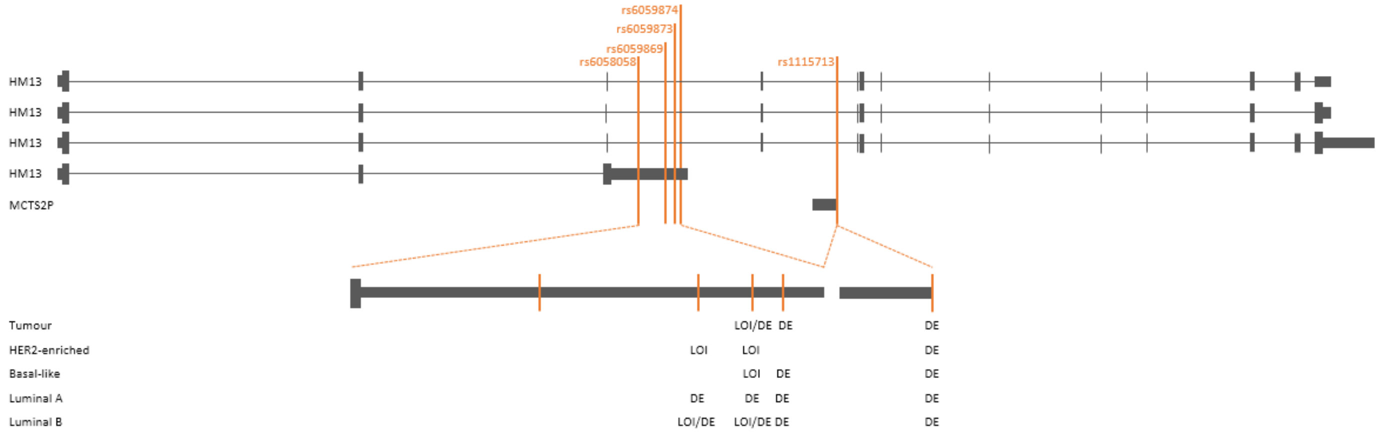

Supplementary Fig. 27 Structure of *HM13/MCTS2P*. Varying transcripts of the *HM13* and *MCTS2P* locus with the imprinted SNPs are shown. Differential expression and LOI information for each SNP in breast cancer (subtypes) is also provided.

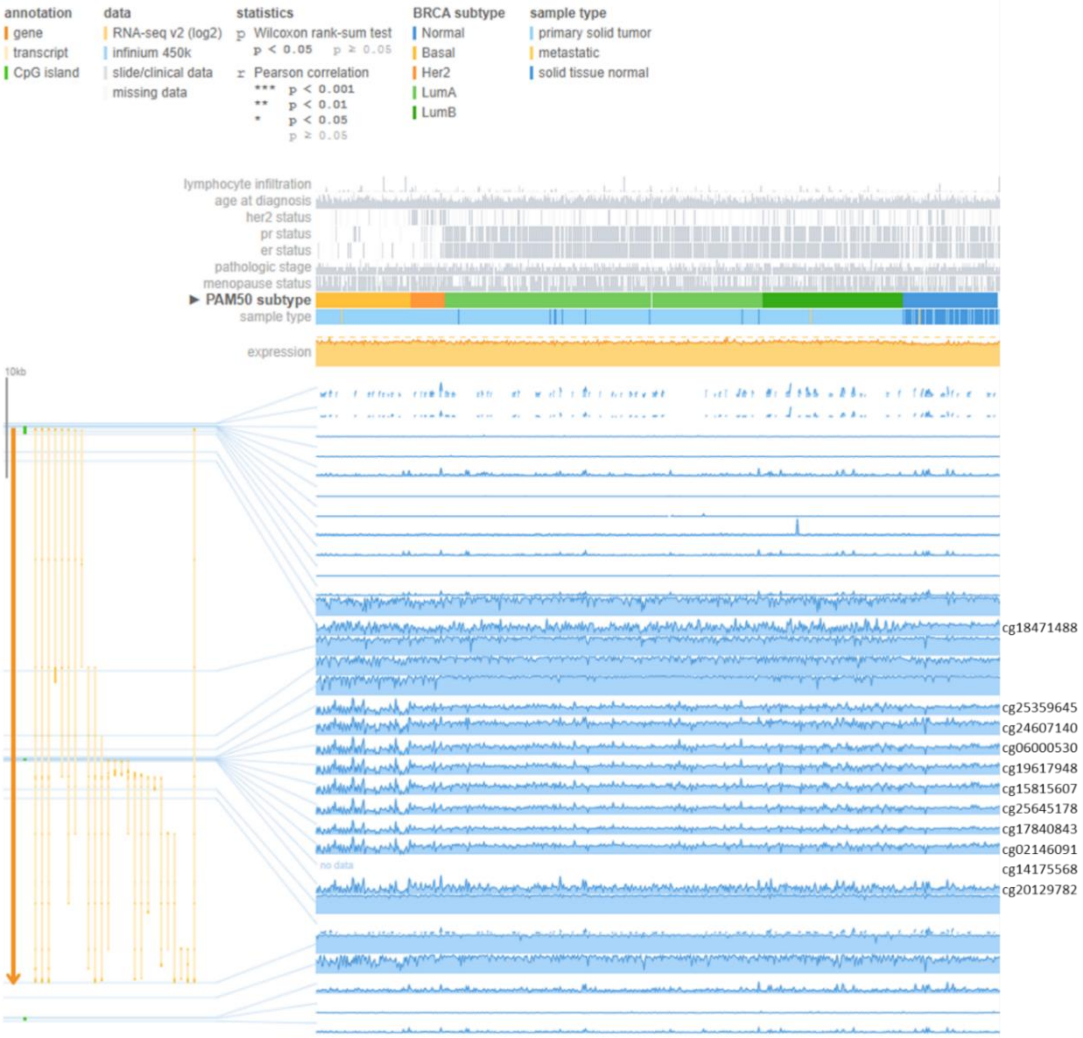

Supplementary Fig. 28 Methylation levels for all probes in TCGA data, probes of interest are denoted with their probe name.

Supplementary Tables

Other Supplementary Tables

Supplementary Table 14 P-values and FDR-adjusted p-values of DI based on the logCPM-value of the least expressed allele count.*

SNP	Gene	p-value	FDR-adjusted p-value
rs3741219	*H19*	0.96627	1
rs2839704	*H19*	0.47665	1
rs2839703	*H19*	0.77801	1
rs10840159	*H19*	0.86182	1
rs2839702	*H19*	0.58448	1
rs2839701	*H19*	0.62859	1
rs2067051	*H19*	0.3955	1
rs2075745	*H19*	0.40713	1
rs2075744	*H19*	0.45442	1
rs2839698	*H19*	0.50549	1
rs2585	*IGF2*	0.99998	1
rs7873	*IGF2*	1	1
rs6059869	*HM13*	0.18073	1
rs6059873	*HM13*	**0.01283**	0.30792
rs8110538	*ZNF331*	0.9785	1
rs8110350	*ZNF331*	0.99327	1
rs7810469	*PEG10*	0.14197	1
rs1053900	*MEG3*	0.99965	1
rs4378559	*MEG3*	0.87254	1
rs12890215	*MEG3*	0.78843	1
rs10863	*MEST*	0.63443	1
rs7582864	*ZDBF2*	0.67573	1
rs3732084	*ZDBF2*	0.42571	1
rs1975597	*ZDBF2*	0.82632	1

*Significant results are shown in bold.

Supplementary Table 15 Differential expression of varying exons in *HM13*. A Wilcoxon Rank Sum test was used to obtain the FDR-adjusted p-values (adjusted p) between tumour and control samples (mean logCPM-values are given in columns mean tumour and mean control and for the breast cancer subtypes). The adjusted p-values for the different subtypes were obtained with a Kruskal-Wallis test (KW) and post-hoc Dunn's test (adjusted p per subtype).

position	mean tumour	mean control	adjusted p	KW	mean BL	adjusted p BL	mean HER2	adjusted p HER2	mean LumA	adjusted p LumA	mean LumB	adjusted p LumB
30102241-30102537	15.30	8.35	4.12E-17	5.71E-20	12.56	1.54E-05	19.77	9.58E-15	13.80	2.79E-12	18.30	1.03E-17
30115287-30115385	14.24	7.87	1.07E-23	6.96E-28	12.31	1.92E-09	18.38	3.32E-21	12.67	9.14E-15	16.81	2.43E-23
30125982-30127596	22.58	14.31	5.29E-17	4.72E-22	19.72	2.37E-06	27.72	1.33E-16	20.35	5.34E-10	26.75	2.05E-19
30132750-30132838	22.65	12.87	2.66E-21	2.17E-26	19.84	5.61E-09	30.00	2.96E-21	19.89	2.11E-12	26.64	2.83E-21
30135185-30136017	4.32	2.83	1.66E-06	4.06E-19	6.61	9.58E-15	4.20	3.47E-05	3.26	9.26E-02	4.78	1.05E-08
30135249-30136917	32.66	18.69	2.47E-24	2.19E-33	36.26	9.34E-21	39.60	1.48E-21	27.15	1.28E-10	37.41	6.73E-24
30137010-30137135	32.51	18.17	2.47E-24	7.29E-30	30.37	2.25E-13	42.07	1.45E-22	28.09	6.52E-13	38.08	5.89E-24
30137867-30137924	17.32	9.38	2.05E-26	2.79E-32	16.55	6.80E-16	22.47	5.89E-24	14.85	7.63E-14	20.18	5.81E-25
30142549-30142632	22.63	12.15	1.24E-26	2.86E-32	22.04	1.12E-16	29.63	6.89E-25	19.36	3.71E-14	25.84	7.51E-24
30147414-30147450	16.79	8.81	2.51E-28	6.12E-34	16.55	2.53E-18	21.99	1.12E-25	14.37	3.37E-15	18.99	1.36E-24
30149437-30149539	34.14	17.37	1.33E-29	3.34E-35	33.96	3.36E-19	44.92	9.93E-27	29.25	3.18E-16	38.11	6.89E-25
30154013-30154098	29.75	14.39	9.88E-33	8.09E-38	29.58	8.52E-21	39.33	2.24E-28	25.71	7.33E-19	32.54	2.20E-26
30155881-30156083	1.76	1.35	1.71E-04	4.72E-11	2.00	7.08E-06	2.13	6.97E-07	1.45	2.28E-01	1.99	2.67E-06
30156923-30157368	75.09	33.83	9.88E-33	3.07E-38	78.64	1.94E-22	99.45	2.18E-28	64.20	1.25E-18	79.09	1.34E-25

Supplementary Table 16 Differential expression of methylation probes in *HM13/MCTSP2* DMR (and exhibiting probable allele-specific methylation) and average methylation over all probes. A Wilcoxon Rank Sum test was used to obtain the FDR-adjusted p-values (adjusted p) between tumour and control samples (mean β-values are given in columns mean tumour and mean control and for the breast cancer subtypes). The adjusted p-values for the different subtypes were obtained with a Kruskal-Wallis test (KW) and post-hoc Dunn's test (adjusted p per subtype).*

probeID**	mean control	mean tumour	adjusted p	KW	mean BL	adjusted p BL	mean HER2	adjusted p HER2	mean LumA	adjusted p LumA	mean LumB	adjusted p LumB
cg02146091	0.43	0.42	0.37	**7.24E-06**	0.328	**9.14E-04**	0.42	0.43	0.45	**0.01**	0.42	0.29
cg06000530	0.42	0.41	0.51	**1.25E-05**	0.304	**6.41E-04**	0.40	0.36	0.44	**0.03**	0.41	0.29
cg15815607	0.45	0.44	0.37	**4.81E-06**	0.335	**1.60E-03**	0.44	0.43	0.48	**0.01**	0.44	0.29
cg17840843	0.31	0.29	0.37	**6.11E-05**	0.217	**3.99E-05**	0.28	0.20	0.31	0.36	0.29	0.34
cg18471488	0.56	0.42	**4.93E-13**	**7.40E-15**	0.494	**1.08E-02**	0.34	**3.3E-07**	0.41	**3.76E-13**	0.43	**1.67E-06**
cg19617948	0.44	0.44	0.37	**3.23E-06**	0.330	**1.18E-03**	0.43	0.43	0.47	**0.01**	0.43	0.29
cg20129782	0.31	0.27	0.31	6.47E-02	0.245	2.51E-03	0.27	0.36	0.28	0.28	0.26	0.25
cg24607140	0.64	0.58	**0.02**	**6.24E-07**	0.464	**1.22E-07**	0.58	0.06	0.61	0.16	0.58	0.25
cg25359645	0.43	0.42	0.37	**1.16E-06**	0.312	**4.55E-04**	0.44	0.36	0.45	**0.01**	0.42	0.29
cg25645178	0.34	0.32	0.37	**2.23E-05**	0.236	**3.59E-05**	0.32	0.22	0.35	0.30	0.33	0.34
Mean(probes)	0.42	0.40	0.74	**2.98E-05**	0.31	**4.87E-05**	0.40	0.13	0.44	0.10	0.40	0.50

*Significant results are shown in bold. **No data was available for probe cg14175568 in TCGA

Supplementary Table 17 Spearman correlation between methylation probes and logCPM-values of exon 30125982-30127596 and *HM13* (full length gene).*

	EXON 30125982-30127596						GENE					
probeID**	adjusted p control	adjusted p tumour	adjusted p all	R control	R tumour	R all	adjusted p control	adjusted p tumour	adjusted p all	R control	R tumour	R all
cg02146091	1	1	1	-0.04	0.02	0.05	1	1	1	0.04	-0.07	0.02
cg06000530	1	1	1	0.13	-0.01	0.03	1	1	1	0.15	-0.10	-0.02
cg15815607	1	1	1	0.07	0.00	0.05	1	1	1	0.15	-0.07	0.04
cg17840843	1	1	1	0.18	0.05	0.04	1	1	0.85	0.00	-0.08	-0.09
cg18471488	1	0.19	**6.1E-05**	-0.04	-0.17	-0.28	1	**0.04**	**1.7E-11**	-0.02	-0.21	-0.42
cg19617948	1	1	1	-0.05	0.01	0.05	1	1	1	0.04	-0.09	0.02
cg20129782	1	1	1	0.11	-0.01	-0.05	1	0.92	0.06	0.11	-0.12	-0.17
cg24607140	0.44	1	1	0.24	0.03	0.01	1	1	0.19	0.16	-0.08	-0.14
cg25359645	1	1	1	0.16	0.06	0.09	1	1	1	0.02	-0.11	-0.01
cg25645178	1	1	1	0.01	0.04	0.01	1	1	0.61	-0.05	-0.09	-0.11
Mean(probes)	0.12	0.85	0.72	0.19	-0.01	-0.02	0.21	**0.04**	**0.02**	0.15	-0.15	-0.14

*Significant results are shown in bold. **No data was available for probe cg14175568 in TCGA

Supplementary Methods

1. Samples & data pre-processing

A total of 113 human healthy control and 506 diseased RNA-seq samples of the TCGA breast invasive carcinoma dataset were downloaded from the TCGA data portal. Downloaded data were already mapped. For all cancer samples, additional expression subtypes based on the PAM50 classifier were obtained from the UCSC cancer genome browser (8 normal-like samples, 92 basal-like, 228 luminal A, 121 luminal B and 57 HER2-enriched). Also, 92 healthy breast samples were download from GTEx and preprocessed in the same way.

After downloading the data, in all samples, variants were called for the non-duplicate uniquely mapped reads with Samtools mpileup/bcftools (v0.1.19) whereby variant sites with a raw read depth lower than 10 in all samples were filtered out. Next, only SNP positions called by Samtools mpileup and present in the public NCBI SNP-archive dbSNP (version 137) were kept. Additionally, loci with only one reference allele in dbSNP (deletions and insertions) and/or loci corresponding with mutations from the Human Gene Mutation Database were filtered out as well[8]. Afterwards, the found SNP positions for all samples were merged and the corresponding nucleotide sequences were determined.

2. Assumptions

It is important to note that several assumptions are made for the methodology described in Methods, Section 4 and 5:

(i) The basic assumption for the detection of imprinting using the proposed methodology is that under the null hypothesis SNP loci are in HWE, which only holds for a panmictic population and for variants that are not under selection, i.e. a single population that is long-term mating.

(ii) In the model developed to screen for imprinted loci, it is assumed that the degree of imprinting is equal in all samples. This translates in the assumption that the cell type composition of each sample is similar.

For the robust measure of imprinting as well as the detection of differential imprinting, samples with the lowest and highest fractions of the alleles with least expression are considered as putatively homozygous resp. heterozygous. Yet, particularly in the case of 100% imprinting, homozygous and heterozygous samples cannot be discriminated using this approach. However, this will not bias the results as (i) in case of 100% imprinting, the robust imprinting criterion will have no practical impact on detection, (ii) to detect differential imprinting, both sample groups are treated equally, implying no bias as long as the genotype frequencies are not associated with the case-control status.

Supplementary References

1. Baran, Y. *et al.* The landscape of genomic imprinting across diverse adult human tissues. *Genome Res.* **25,** 927–936 (2015).
2. Joshi, R. S. *et al.* DNA Methylation Profiling of Uniparental Disomy Subjects Provides a Map of Parental Epigenetic Bias in the Human Genome. *Am. J. Hum. Genet.* **99,** 555–566 (2016).
3. Jurtle, R. Geneimprint : Home. Available at: http://www.geneimprint.com/. (Accessed: 24th August 2017)
4. Morison, I. M., Paton, C. J. & Cleverley, S. D. The imprinted gene and parent-of-origin effect database. *Nucleic Acids Res.* **29,** 275–6 (2001).
5. Steyaert, S. *et al.* SNP-guided identification of monoallelic DNA-methylation events from enrichment-based sequencing data. *Nucleic Acids Res.* **42,** e157 (2014).

6. Laddha, S. V, Ganesan, S., Chan, C. S. & White, E. Mutational landscape of the essential autophagy gene BECN1 in human cancers. *Mol. Cancer Res.* **12,** 485–90 (2014).

7. Frost, J. M. *et al.* Evaluation of Allelic Expression of Imprinted Genes in Adult Human Blood. *PLoS One* **5,** e13556 (2010).

8. Stenson, P. D. *et al.* The Human Gene Mutation Database: building a comprehensive mutation repository for clinical and molecular genetics, diagnostic testing and personalized genomic medicine. *Hum. Genet.* **133,** 1–9 (2014).

9. Molenberghs, G. & Verbeke, G. Likelihood Ratio, Score, and Wald Tests in a Constrained Parameter Space. *Am. Stat.* **61,** 22–27 (2007).
