# Supplementary material for: A comprehensive overview of genomic imprinting in breast and its deregulation in cancer
Source: Nat Commun. 2018 Oct 8;9:4120. doi: 10.1038/s41467-018-06566-7 (PMC6175939; doi:10.1038/s41467-018-06566-7)
Supplement: Supplementary file 3 — Description of Additional Supplementary Files [file 41467_2018_6566_MOESM3_ESM.pdf]

Description of Additional Supplementary Files

File Name: Supplementary Data 1
Description: Parameters of all SNPs in gene HM13. The dbSNP IDs (dbSNP), likelihood ratio test statistic (LRT), estimated degrees of imprinting (i), p-values of the LRT (p), allele frequencies (pA), estimated sequencing error rates (SE), mean coverage (cov), the number of samples (nr_samples), the GOF-likelihood value (GOF; eliminates artefacts, but may also exclude transcripts with mixed imprinting patterns), the χ^2 p-value for the symmetry test (sym; eliminates loci featured by allelespecific expression), the median imprinting value (med. imp.; retains robustly imprinted results), the genomic annotation (annotation) and the reason why a SNP was filtered (filtered) are shown for all SNPs. Green indicates the values that passed our filters and in red the SNPs detected as imprinted by our method are shown.

File Name: Supplementary Data 2
Description: Parameters of all SNPs in genes for which no consistent imprinting results were found. The dbSNP IDs (dbSNP), likelihood ratio test statistic (LRT), estimated degrees of imprinting (i), p-values of the LRT (p), allele frequencies (pA), estimated sequencing error rates (SE), mean coverage (cov), the number of samples (nr_samples), the GOF-likelihood value (GOF; eliminates artefacts, but also indicates transcripts with mixed imprinting patterns), the χ^2 p-value for the symmetry test (sym; eliminates loci featured by allele-specific expression), the median imprinting value (med. imp.; retains robustly imprinted results) and the reason why a SNP was filtered (filtered) are shown for all SNPs. The last column (imprinting status/annotation/…) indicates the genomic annotation and whether or not it was filtered/imprinted. Green indicates the values that passed the filters and in red the SNPs detected as imprinted by our method are shown.

File Name: Supplementary Data 3
Description: DNA methylation in TCGA breast samples. Probe id (ID), the gene in which it is located (Gene), if it was linked with a SNP (dbSNP), number of control and tumour samples (nr_control and nr_tumour, respectively), the median methylation levels (Median control and Median tumour) and whether or not the probe is located in a DMR (DMR?) are given. The FDR-adjusted p-values of the Wilcoxon Rank Sum Test to compare methylation levels of control data with tumour data are shown in the last column (adjusted p). Significant results are shown in red and hemimethylated probes (methylation between 0.33 and 0.66) are coloured light red.

File Name: Supplementary Data 4
Description: FDR-adjusted p-values of DI analysis for all SNPs. Tumour samples as well as the varying subtypes were compared to the normal samples. Significant results are shown in red.

File Name: Supplementary Data 5
Description: FDR-adjusted p-values and log fold changes of differential expression (DE) analysis. CPM-values of the SNPs were used to compare control versus diseased samples. Differential expression between control and tumour was analysed with a Wilcoxon Rank Sum test, while analysis per subtype was done with a Kruskal-Wallis test and Dunn's post-hoc test. Significant DE is coloured in red

File Name: Supplementary Data 6
Description: Contingency tables for differential expression (DE) vs copy number variation (CNV) of each imprinted gene in tumour data. FDR-adjusted p-values for imprinted genes of linear models for expression (dependent variable) and copy number variation are also provided in column "p linear model".
